# PARP Inhibitors as Radiosensitizers: Current Evidence and Future Directions

**DOI:** 10.1007/s11912-026-01800-8

**Published:** 2026-06-01

**Authors:** Eurico Pereira, Filipa Pereira, Gabriela Campos, Ana M. Abrantes, Maria F. Botelho, Pedro Silva-Vaz, Ana S. Pires

**Affiliations:** 1 Coimbra Institute for Clinical and Biomedical Research (iCBR) area of Environment Genetics and Oncobiology (CIMAGO), Institute of Biophysics, Faculty of Medicine, Azinhaga de Santa Comba, Pólo III - Pólo das Ciências da Saúde, University of Coimbra, Coimbra, 3000-548 Portugal; 2 Center for Innovative Biomedicine and Biotechnology (CIBB), University of Coimbra, Rua Larga, Coimbra, 3004-504 Portugal; 3 Clinical Academic Center of Coimbra (CACC), Praceta Professor Mota Pinto, 3004-561 Coimbra, Portugal; 4 General Surgery Department, Unidade Local de Saúde (ULS) de Coimbra, Praceta Professor Mota Pinto, Coimbra, 3000-075 Portugal

**Keywords:** Radiotherapy, Poly(ADP-ribose) Polymerase inhibitors, Combined modality therapy, DNA repair, Radiosensitization

## Abstract

**Purpose of Review:**

Radiotherapy remains a fundamental pillar of cancer treatment, however its efficacy is still often limited by tumor radioresistance and toxicity to surrounding healthy tissues. Poly(ADP-ribose) polymerase inhibitors (PARPi) have emerged as potent radiosensitizers by impairing DNA repair mechanisms, particularly base excision repair, thereby enhancing the cytotoxic effects of ionizing radiation (IR). This review provides a critical narrative synthesis, based on a structured literature search, consolidating current preclinical and clinical evidence on the therapeutic potential of combining PARPi with various IR modalities, including X-rays, γ-rays, α- and β-particles, protons, and carbon ions, across a broad spectrum of tumor types.

**Recent Findings:**

The findings reveal that PARPi consistently enhance radiosensitivity, with the magnitude of effect influenced by radiation type, tumor-specific DNA repair capacity, and PARPi pharmacodynamics. Notably, combinations with high-LET radiation (*e.g.*, carbon ions) demonstrate superior efficacy in certain contexts, while emerging data highlights the immunomodulatory potential of PARPi plus IR strategies via cGAS-STING pathway activation. Clinical trials confirm the feasibility of these combinations, although toxicity profiles vary by tumor type and treatment regimen.

**Summary:**

This review underscores the promise of PARPi plus radiotherapy combinations and identifies key avenues for future research, including biomarker-driven patient stratification and the development of triple-combination strategies to optimize therapeutic outcomes.

**Supplementary Information:**

The online version contains supplementary material available at 10.1007/s11912-026-01800-8.

## Introduction

Cancer remains a major global health challenge, accounting for a significant proportion of morbidity and mortality worldwide. In 2022, cancer was responsible for approximately 20 million new cases and 9.7 million deaths worldwide, with these incidence and mortality rates varying by region, gender, and cancer type, and with a rising burden in both developed and developing countries [1–3]. According to Globocan 2022 Cancer Tomorrow, the cancer incidence and mortality will increase 63.4% and 73.3% by 2045, respectively [1, 4, 5]. Furthermore, the diversity of cancer types, each with unique biological characteristics and therapeutic challenges, further complicates effective management and underscores the need for tailored treatment strategies [6–8].

Radiotherapy is a fundamental modality in cancer therapy, used in about half of all cancer patients [9–12]. It can employ different types of ionizing radiation (IR), as photons or particles such as α- and β-particles, protons, electrons, neutrons, and carbon ions, each with distinct physical and biological properties [13–16]. Low linear energy transfer (LET) radiation, such as photons, induces DNA damage mainly through sparsely ionizing events, while high-LET radiation (*e.g.*, carbon ions, α-particles) causes more complex and clustered DNA lesions, leading to increased cell killing [13–18]. The therapeutic effect of IR is mediated by both direct DNA damage and indirect effects via reactive oxygen and nitrogen species [13–17]. However, the efficacy of radiotherapy is limited by the intrinsic DNA repair capacity of tumor cells, the development of radioresistance, and the risk of toxicity to surrounding healthy tissues [13–17, 19, 20].

Poly (ADP-ribose) polymerase (PARP) is a family of nuclear proteins that plays a fundamental role in DNA repair and in maintaining genome integrity [21]. PARP1 stands out as the most extensively recognized and characterized member of this family, which acts in DNA repair pathways [22]. PARP1 plays a crucial role in the base excision repair (BER) pathway and, consequently, in the repair of single-strand breaks (SSB) [23]. Upon detecting and recognizing SSB via its DNA-binding domain, the protein catalyzes PARylation by cleaving NAD⁺ and synthesizing PAR chains that scaffold the damage site and recruit other essential proteins in BER [22, 24–29]. Despite the widely known function of PARP1 in BER, there is evidence that PARP1 also acts in the repair of double-strand breaks (DSB) through the recruitment of repair enzymes in homologous recombination repair (HRR), such as RAD51, and in the inactivation of DNA-dependent protein kinases in non-homologous end joining (NHEJ) pathway [24–27, 30, 31]. PARP inhibitors (PARPi) work by inhibiting the enzymatic activity through two mechanisms: (1) binding to the catalytically active site of PARP, competing with NAD^+^, or (2) trapping PARP in the DNA molecule at the damaged site, preventing it from carrying out the PARylation [31]. Thus, PARPi can block the BER pathway, impeding the repair of SSB. This results in the accumulation of unrepaired SSB and the subsequent formation of DSB, leading to the collapse of the replication fork, and ultimately cell death, especially in cells deficient in HRR, such as those with BRCA1/2 mutations [24–27]. This forms the basis of synthetic lethality, where simultaneous impairment of two DNA repair pathways results in selective tumor cell death [24, 25, 27, 32].

**Fig. 1:**
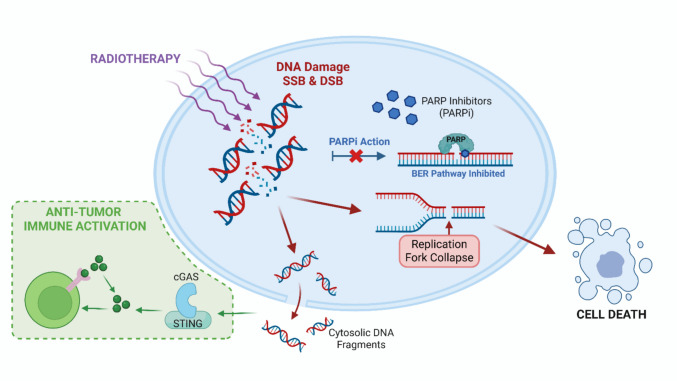
Mechanism of Radiosensitization by PARPi Combined with Radiotherapy. This figure illustrates the molecular mechanisms underlying the synergy between radiotherapy and PARPi. Radiotherapy induces DNA damage, including SSB and DSB. PARPi blocks the BER pathway, preventing the repair of SSB and leading to the accumulation of DSB during replication. The accumulation of unrepaired DSB results in cell death through replication fork collapse and mitotic catastrophe. Additionally, increased cytosolic DNA activates the cGAS-STING pathway, triggering antitumor immune activation. This mechanism explains the radiosensitizing effect of PARPi and highlights their potential to enhance radiotherapy efficacy while modulating the tumor microenvironment. Created in BioRender. Pereira, E. (2026) https://BioRender.com/jajmn5v

Combining PARPi with IR is a promising strategy to enhance tumor radiosensitivity. IR induces extensive DNA damage, including SSB and DSB. Under normal conditions, SSB are rapidly repaired via the BER pathway, which depends on PARP activity. PARPi block this pathway, preventing the repair of SSB and causing their persistence. During DNA replication, these unrepaired SSB are converted into DSB, which are far more cytotoxic. The accumulation of DSB leads to replication fork collapse, chromosomal instability, and ultimately cell death. Beyond direct cytotoxicity, PARPi plus IR also promotes immune activation: increased cytosolic DNA fragments activate the cGAS–STING pathway, triggering type I interferon signaling and enhancing antitumor immunity. This dual mechanism, DNA repair inhibition and immune modulation, explains the radiosensitizing effect of PARPi and highlights their potential to improve radiotherapy efficacy while reshaping the tumor microenvironment [14, 20, 24, 33–35]. Figure 1 summarizes the mechanistic rationale for combining PARPi with radiotherapy, highlighting DNA repair inhibition and immune activation pathways.

Given the potential synergistic interaction between PARPi and IR, ongoing research is exploring this combination across various cancer types. Accordingly, this review aims to consolidate current evidence on the therapeutic application of PARPi in combination with different forms of IR for cancer treatment.

This work is a narrative critical review supported by a structured and systematic literature search, rather than a formal systematic review or meta-analysis. While PRISMA-inspired elements were used to improve transparency in study identification and selection, no formal risk-of-bias assessment or quantitative synthesis was performed. All methodological details, including the search strategy (Online Resource 1), eligibility criteria, and PRISMA flow diagram (Online Resource 2), are provided in the Supplementary Information. Briefly, the search identified 2190 records, of which 119 studies were included, encompassing multiple radiation modalities, tumor types and experimental settings. Data extracted from each selected article is gathered and summarized in Online Resource 3. The studies were grouped and discussed according to the type of radiation, namely X-rays, γ-rays, α-particles, β-particles, protons, and carbon ions, as well as according to the type of study, *i.e. *in vitro study, in vivo study, or clinical trial.

## X-Rays

X-rays are a form of electromagnetic radiation. Their high energy and short wavelength enable significant penetration through biological tissues and various materials. X-rays interaction with matter is characterized by their ability to ionize atoms and molecules, primarily through the ejection of electrons, which underpins both their diagnostic utility and potential for biological damage [15, 16, 36, 37]. A key physical property of X-rays is their classification as low-LET radiation, with LET values typically less than 10 keV/μm [36, 38]. Biologically, X-rays induce damage through both direct and indirect mechanisms. Direct effects involve the ionization and breakage of DNA strands, while indirect effects are mediated by reactive oxygen and nitrogen species generated from water radiolysis [15, 39, 40]. Low-LET radiation deposits energy sparsely along its path, resulting in predominantly indirect biological effects mediated by the generation of reactive oxygen and nitrogen species. The biological consequences include SSB and DSB, chromosomal aberrations, and, at higher doses, cell death. Compared to high-LET radiation, low-LET X-rays generally induce less complex DNA damage, which is more amenable to cellular repair mechanisms [15, 16, 36–38, 41].

Several in vitro studies have evaluated the combination of PARPi (Olaparib, Niraparib, Rucaparib, Veliparib, Talazoparib, among others) with X-ray irradiation across a wide range of tumor models, such as breast [42–46], ovarian [47, 48], glioblastoma [49–56], pancreatic [50, 57–60], lung [59, 61–67], prostate [50, 68–70], head and neck cancers [61, 71–76], among others [57, 77–99]. Most studies, although differing in tumor type and the specific PARPi employed, consistently reported enhanced radiosensitivity compared to radiation alone. This increased sensitivity was attributed to synergistic cytotoxic effects, reflected in reduced tumor cell proliferation, increased DNA damage, and the induction of cell cycle arrest and apoptosis. In oral squamous cell carcinoma models, Yu et al*.* further expanded this mechanistic understanding by showing that Olaparib combined with X-rays not only enhanced radiosensitivity but also reduced tumor cell migration and invasion through downregulation of IL-17A and inhibition of the NF-κB and p38 MAPK pathways, which are known to promote inflammatory and pro-metastatic responses [72]. However, some evidence indicates that the efficacy of PARPi plus radiotherapy combinations may depend on specific tumoral or cellular factors. For example, in colon cancer, Romeo et al*.* observed that the combination was effective only in cells lacking TP53 mutations [78]. Conversely, in diffuse midline glioma, H3K27M-mutant cells, which exhibit impaired homologous recombination repair, showed greater sensitivity to Olaparib and X-rays compared with isogenic controls [89]. Overall, PARPi plus radiotherapy combinations demonstrate radiosensitizing activity across multiple tumor models, prompting further validation in in vivo models and clinical studies.

Following the promising in vitro results, several in vivo studies have tested the combination of PARPi and X-rays in animal models [42, 43, 46, 47, 49, 50, 57, 61–65, 68, 69, 71, 72, 74, 80–85, 87–95, 99–110]. These studies employed the same tumor types and PARPi compounds as those investigated in the in vitro experiments. The results consistently demonstrated that the combination delays tumor growth, induces cell cycle arrest and apoptosis, and prolongs survival more effectively than either radiation or PARPi alone. Beyond these cytotoxic effects, some authors expanded the mechanistic understanding of PARPi plus radiotherapy interaction in various tumor types (hepatocellular carcinoma, diffuse midline glioma, colorectal cancer and lung cancer) revealing a strong immunomodulatory component [89, 90, 108, 110]. Specifically, combined treatment was shown to activate the cGAS–STING pathway, a key cytosolic DNA-sensing mechanism, resulting in robust type I interferon production and increased recruitment of natural killer and cytotoxic T lymphocytes to the tumor microenvironment [89, 90, 108, 110]. These immune responses contributed not only to improve local tumor control but also to abscopal effects, characterized by regression of distant, non-irradiated lesions, suggesting systemic antitumor immunity triggered by the combination therapy [90]. This systemic response was markedly stronger in immunocompetent compared with immunodeficient mice, indicating that a functional immune system is essential for mediating these effects [90]. Despite these encouraging results regarding PARPi plus X-rays combinations, exceptions have been reported. Two studies found that combining Olaparib with X-rays did not yield therapeutic efficacy in pancreatic cancer models [111, 112]. However, these findings contrast with those reported by Meng et al., who observed a positive radiosensitizing effect in the same tumor type when using Veliparib, suggesting that in some specific tumors, the outcome may be PARPi-dependent [57]. While PARP inhibition generally enhances tumor control in combination with X-rays, two in vivo studies have also reported adverse effects in healthy tissues. For instance, Lourenco et al. observed significant body mass loss and increased damage to the esophagus and skin in irradiated mice, and more recently, Jeong et al. demonstrated in healthy intestinal models that Olaparib can exacerbate radiation-induced intestinal injury, increasing apoptosis and structural damage [106, 113]. Altogether, these in vivo results confirm the therapeutic promise of combining PARPi with X-rays but also point to the need to define the maximum tolerated dose that achieves tumor control without causing unacceptable toxicity, an issue subsequently addressed in early-phase clinical trials.

Considering the results reported so far about the combination of PARPi with X-ray therapy, several phase I clinical trials have been conducted to determine the maximum tolerated dose and assess potential adverse events of Olaparib, Veliparib, Niraparib, or Rucaparib administered concurrently with X-rays. These trials included patients with a variety of tumor types, such as triple-negative breast cancer [114, 115], inflammatory or locoregionally recurrent breast cancer [116], brain metastases [117], advanced ovarian or fallopian tube cancer with peritoneal carcinomatosis [118, 119], esophageal cancer [120], head and neck squamous cell carcinoma [121] and advanced epithelial ovarian cancer [122]. In triple-negative breast cancer, Olaparib was safely combined with X-rays, with no dose-limiting or severe late toxicities observed, and promising survival outcomes were reported [114, 115]. In contrast, patients with inflammatory or locoregionally recurrent breast cancer experienced more severe acute and late toxicities, highlighting that safety profiles can vary depending on tumor subtype and prior treatment history [116]. Among other cancers, including esophageal cancer and head and neck squamous cell carcinoma, higher doses of Olaparib were associated with dose-limiting toxicities, whereas lower doses were generally well tolerated, indicating a clear relationship between administered dose and toxicity [120, 121]. The combination of Veliparib with X-rays has also been investigated in multiple contexts. In patients with brain metastases, manageable grade 3/4 adverse events such as fatigue, hypokalemia, and hyponatremia were reported, with median survival exceeding expectations, suggesting potential efficacy even in challenging clinical scenarios [117]. Similarly, in advanced ovarian or fallopian tube cancer with peritoneal carcinomatosis, Veliparib plus X-rays resulted in prolonged disease stability, with gastrointestinal symptoms, fatigue, and myelosuppression being the most frequent toxicities, demonstrating the feasibility of this approach across diverse tumor types [118, 119]. Overall, these studies indicate that PARPi can be combined with X-rays with manageable toxicity, although safety and efficacy outcomes vary depending on tumor type, prior treatment history and administered PARPi dose.

Emerging preclinical studies in rectal, cervical and lung cancers have explored triple-combination strategies to further enhance the efficacy of PARPi plus radiotherapy regimens [64, 87, 88]. These approaches include the addition of MEK inhibitors, chemoradiotherapy, thermoradiotherapy, or antibody-modified nanoparticles targeting Bifidobacterium (a bacteria which preferentially colonize hypoxic tumor regions) to the PARPi plus radiotherapy regimens. In vivo, these strategies have demonstrated enhanced tumor growth inhibition and prolonged survival in mice, often with manageable toxicity [64, 87, 88]. Although still experimental, these findings suggest potential avenues for optimizing PARPi plus radiotherapy combinations in future studies.

## γ-Rays

γ-rays are high-frequency electromagnetic waves originating from nuclear transitions. Occupying the uppermost region of the electromagnetic spectrum, γ-rays have a deep penetration and significant ionization potential through matter [15, 16, 37, 38, 123]. A defining physical parameter of γ-rays is their low-LET, generally less than 10 keV/μm. As low-LET radiation, γ-rays deposit energy sparsely along their tracks, producing isolated ionization events. This contrasts with high-LET radiations (*e.g.*, alpha particles, heavy ions), which deposit energy densely and over shorter distances [14–16, 37, 38, 123]. Biologically, γ-rays induce both direct and indirect damage. Direct effects involve ionization of DNA molecules, leading to SSB and DSB, while indirect effects arise from the water radiolysis, generating reactive oxygen and nitrogen species that further damage cellular components. Due to the sparsely ionizing nature of γ-rays, the indirect damage dominates the biological effect, contributing 70–90% of the total DNA strand breaks. The low-LET nature of γ-rays results in a predominance of isolated DNA lesions, which are generally more amenable to cellular repair mechanisms [14–16, 37, 38, 123].

To assess whether PARP inhibition enhances radiosensitivity to γ-rays, several in vitro studies have been conducted across multiple tumor types, including lung [101, 104, 105, 124–126], breast [127, 128], prostate [102, 129, 130], liver [131] and pancreatic cancer [125, 132, 133], among others [100, 107, 134–145]. A variety of PARPi were tested across these studies, with Olaparib, Veliparib, Rucaparib, and Talazoparib being the most frequently employed. Despite methodological differences, the findings were largely consistent, demonstrating significant radiosensitization in most cancer cell lines tested. For instance, in LoVo colorectal cancer cells, treatment with the PARPi AG14361 in the post-irradiation recovery period significantly reduced cell survival compared with non-treated controls. In fact, within 24 h, AG14361 suppressed the repair and recovery processes typically activated after γ-ray exposure by approximately 73%, leading to a marked decrease in viable cells [101]. An exception was reported by Guillot et al*.*, who observed that only four out of seven liver cancer cell lines were sensitive to Veliparib (2 h treatment), with enhanced radiosensitivity detected exclusively in HepG2 and PLC-PRF-5 cells [131]. The authors proposed that the efficacy of this combined treatment may depend on the intrinsic DNA repair capacity of each cell line, suggesting that assessing DNA repair proficiency in tumors from patients enrolled in PARPi-based clinical trials could help optimize radiotherapy regimens [131].

Several in vivo studies have investigated the synergistic effects of PARP inhibition combined with γ-ray irradiation across various tumor models, including lung [124–126, 146], breast [146], pancreatic [125], glioblastoma [136, 141, 147], nasopharyngeal carcinoma [143] and pediatric high-grade astrocytomas [144]. Overall, these studies consistently showed that the combined treatment increased tumor growth delay and extended median survival compared with either treatment alone. In glioblastoma models, however, radiosensitization appeared to depend on the specific PARPi used. Two studies reported that Olaparib enhanced radiosensitivity [136, 141], whereas a study using Veliparib in combination with γ-radiation found no radiosensitizing effect in GBM12 cells [147]. These findings suggest that the outcome of PARPi combined with γ-radiation may vary depending on both the inhibitor and the intrinsic characteristics of the tumor cells. In addition to these findings, Albert et al*.* demonstrated that pre-treatment with Veliparib before irradiation reduced tumor vessel formation in mice, indicating that this approach may also impact tumor angiogenesis [126]. Collectively, these findings demonstrate the radiosensitizing potential of PARPi when combined with γ-radiation, but also highlight considerable variability between tumor models, underscoring the need for mechanistic studies to clarify the determinants of treatment response.

## α-Particles

Αα-particles are composed by two protons and two neutrons and are predominantly emitted during the radioactive decay of heavy nuclei. Due to their relatively large mass and charge, α-particles exhibit a short penetration depth in biological tissues, typically less than 100 μm, while depositing energy densely along their path, resulting in a high ionization density [15, 16, 37, 38]. Therefore, a defining characteristic of α-particles is their high-LET, generally around 100 keV/μm [15–17, 37, 148, 149]. The production of dense ionization tracks leads to complex, clustered DNA damage that is difficult for cells to repair. This leads to efficient induction of cell death, particularly through apoptosis and mitotic catastrophe, and underpins the therapeutic potential of α-emitting radionuclides in targeted cancer therapies [15–17, 37, 148–150].

To date, only Mass et al*.* have investigated the effects of alpha radiation combined with PARP inhibition in vitro [151]. Specifically, they used an α-emitter compound, ^223^RaCl_2_ (radium-dichloride) with the PARPi Olaparib in prostate cancer cell line, PC-3. The combination resulted in a modest, dose-dependent reduction in cell survival and a slight increase in DNA damage. Overall, the results were limited in magnitude, suggesting limited therapeutic potential for this approach. However, given that evidence is currently restricted to a single in vitro study in one tumor type, further research is required to determine whether these findings can be reproduced or optimized in other experimental settings.

## β-Particles

β-particles are high-speed electrons emitted during the radioactive decay of unstable atomic nuclei, a process known as beta decay. Unlike monoenergetic alpha decay, beta particles are polyenergetic because the decay energy is shared between the beta particle and a antineutrino, meaning the average energy is typically about one-third of the maximum energy [15, 16, 37]. As charged particles, beta particles have a finite range and are generally less penetrating than uncharged photons. While the range depends on their energy, high-energy beta emitters used in therapy have a mean penetration depth in tissue around 4.1 mm and a maximum of 11 mm. A defining physical characteristic of beta particles is their low-LET, meaning that beta particles deposit energy sparsely along their tracks, resulting in a more uniform and less dense ionization pattern compared to high-LET radiation, such as alpha particles [15, 16, 37, 38, 152, 153]. Biologically, the low-LET nature of beta particles results in the indirect action dominating the cellular damage (accounting for up to 70–90% of total DNA strand breaks), primarily through the generation of reactive oxygen and nitrogen species via water radiolysis. Direct effects involve ionization of DNA, leading to SSB and a significantly lesser extent of DSB [15, 16, 37, 38, 152, 153].

A series of in vitro studies have investigated the effects of combining β-particle radiation with PARPi across various tumor types [52, 60, 130, 138, 154, 155]. These analyses include: ^131^I with A-966492 in glioblastoma cells [155]; ^177^Lu-DOTA-TATE with Olaparib in multiple myeloma, pancreatic carcinoma, colon adenocarcinoma, melanoma and osteosarcoma [60, 154]; ^131^I-tositumomab with Olaparib in lymphoma [138]; ^192^Ir with Rucaparib and ^177^Lu-DOTA-TATE with Olaparib in prostate cancer [130, 151]; ^131^I-MIBG with Rucaparib or Olaparib in neuroblastoma and glioblastoma [52]; and ^177^Lu-DOTA-TOC with Olaparib or Rucaparib in small cell lung cancer [156]. Overall, these studies demonstrated that PARPi significantly enhanced the efficacy of IR in the tested cell lines, possibly by promoting significantly higher amounts of persistent DNA damage and decreasing cell survival. The only exception refers to the combination of Olaparib with 1⁷⁷Lu-DOTA-TATE in COLO-677 and EJM multiple myeloma cell lines, where no additional reduction in cell survival was observed compared to radiation alone, suggesting a lack of radiosensitizing effect of Olaparib in this specific cellular context [60]. Overall, these findings demonstrate that PARPi enhances the cytotoxic effects of β-particle irradiation in most tumor models tested, although the magnitude of radiosensitization appears to vary depending on the cellular context and the specific PARPi used.

In vivo studies in mice have further supported the potential of PARPi as radiosensitizers for β-particles radiotherapy [156–158]. For instance, Bao et al*.* and Rauch et al*.* demonstrated the combination between PARPi and ^177^Lu-DOTAGA.(SA.FAPi)_2_ or ^177^Lu-DOTA-TOC in mice with triple-negative breast cancer or lung cancer significantly delayed tumor growth with tolerable toxicity and extended median survival in the combination groups [156, 158]. Similarly, Feijtel et al*.* investigated the effect of Olaparib combined with ^177^Lu-DOTA-TATE in mice with metastasized neuroendocrine tumors [157]. Their findings revealed an improved therapeutic efficacy in CA20948 tumors, but not in NCI-H69 tumors, highlighting a tumor type-dependent response to this combination. Notably, the only ex vivo study evaluating the same combination in a pancreatic neuroendocrine tumor model [154] reported increased DNA DSB, indicating that Olaparib can also radiosensitize these cancer cells to ^177^Lu-DOTA-TATE. Collectively, these in vivo studies confirm the therapeutic potential of PARP inhibition in β-particle radiotherapy and suggest that tumor type may modulate treatment efficacy.

Regarding the use of PARPi in combination with β-particles, to date, only Hallqvist et al*.* have evaluated the combination of Olaparib with the β-emitter 1⁷⁷Lu-DOTA-TATE in a phase 1 clinical trial involving patients with somatostatin receptor–positive tumors [159]. The study demonstrated the feasibility and tolerability of this combination, with toxicity primarily related to thrombocytopenia. The observed bone marrow toxicity underscores the need for careful hematologic monitoring [159]. Further randomized trials are warranted to determine whether this approach provides clinical benefit over ⁷⁷Lu-DOTA-TATE monotherapy.

Additionally, Delbart et al*.* and Schaefer et al*.* directly compared the synergetic effects of Olaparib with three types of radiation, β-particles (^177^Lu-DOTA-TATE or ^131^I-tositumomab), X-ray and γ-rays, in multiple myeloma, pancreatic carcinoma, colon adenocarcinoma, melanoma and lymphoma cell lines [60, 138]. Their results demonstrated that, across all tested cell lines, Olaparib exerted a stronger radiosensitizing effect when combined with X-rays and γ-rays than with β-particles. These findings indicate that, although PARPi enhances the efficacy of β-particle radiotherapy, alternative radiation modalities may offer even greater therapeutic potential for these specific cancers [60, 138]. However, for prostate cancer the results were different, as the β-emitter ^177^Lu combined with Olaparib demonstrated a higher radiosensitization efficacy compared to the α-emitter [151]. The increased cytotoxicity observed with 1⁷⁷Lu is mainly due to its low LET characteristics, which lead to a higher frequency of DNA SSB. This makes the inhibition of base excision repair by PARPi particularly effective; however, the efficacy of this combination seems to depend on the tumor type. These comparative analyses highlight that, while PARPi enhances the efficacy of β-particle therapy, combinations with X- and γ-rays have generally shown stronger radiosensitizing effects in certain tumors.

## Protons

Protons are positively charged particles originated from atomic nuclei. Their unique physical properties enable precise energy deposition in matter, most notably characterized by the formation of their characteristic Bragg Peak, where protons deposit the majority of their energy at a specific depth, minimizing dose to surrounding healthy tissues [14–16, 37, 160]. This results in a highly localized and variable ionization pattern along the proton track: low ionization (low-LET) at the entrance and a sharp increase to high-LET near the Bragg Peak. The LET is a critical parameter in proton therapy, as it directly influences the pattern and complexity of biological damage [14–16, 37, 160–162]. The variation in LET along the proton path has profound implications for biological effectiveness. At low-LET regions (entrance), protons induce DNA damage patterns similar to photons, primarily causing isolated DNA lesions. As LET increases near the Bragg Peak, the density of ionization events rises, leading to more complex DNA damage, including clustered DSB that are more challenging for cellular repair mechanisms [14–16, 37, 162–164]. This complexity underpins the concept of variable Relative Biological Effectiveness (RBE), which quantifies the biological potency of protons relative to reference radiation (typically photons). While a constant RBE of 1.1 is often used clinically, substantial evidence demonstrates that RBE increases with LET, particularly at and beyond the Bragg Peak, potentially reaching values significantly higher than 1.1 and contributing to both enhanced tumor control and increased risk of normal tissue toxicity [14–16, 37, 160, 162–167]. The interplay between LET and RBE is central to optimizing proton therapy. High-LET regions near the Bragg Peak are associated with increased biological damage and RBE, necessitating careful treatment planning to balance tumor eradication with the minimization of adverse effects in normal tissues. Advances in microdosimetry and modeling continue to refine our understanding of LET-dependent RBE, supporting the development of more personalized and effective proton therapy regimens [15, 16, 37, 160, 162, 164–166].

Several in vitro studies have explored the synergistic effect of PARP inhibition combined with proton irradiation across different tumor models, including lung cancer [59, 66, 168], pancreatic cancer [59, 66, 112, 168], chondrosarcoma [97], esophageal carcinoma [169], medulloblastoma [170], head and neck squamous cell carcinoma [75, 76], breast cancer [46, 171] and osteosarcoma [171]. Among the PARPi tested, Olaparib was the most frequently used [59, 66, 97, 112, 168, 169]; however, other authors employed Pamiparib, Talazoparib, Niraparib, and Veliparib [46, 75, 76, 170, 171]. Collectively, the studies support that targeting PARP can effectively radiosensitize cancer cells to proton therapy, leading to persistent DNA damage, extensive cell killing, and tumor growth delay. However, the extent of DNA damage and the subsequent cellular response to proton irradiation appear to be LET-dependent. According to Han et al*.*, under low LET conditions (entrance protons), PARPi induces replication fork stalling, allowing DNA damage recognition and repair [171]. In contrast, high LET protons at the Bragg Peak generate complex DNA lesions that replication forks bypass in the presence of PARPi, leaving behind single-stranded DNA gaps that contribute to genomic instability and enhanced cytotoxicity [171]. Overall, these in vitro studies demonstrate that PARPi can enhance the cytotoxic effects of proton therapy, supporting further evaluation of this combination in in vivo models.

Building on these in vitro findings, subsequent in vivo studies were conducted to validate the radiosensitizing potential of PARPi in preclinical tumor models. Waissi et al*.* and Ben Kacem et al*.* evaluated the effect of pre-treatment with Olaparib before proton therapy in mice with pancreatic ductal adenocarcinoma and breast cancer [46, 112]. The results demonstrated that this combination enhanced progression-free survival and significantly delayed tumor growth, compared to proton therapy alone. Similar results were obtained by Simovic et al*.* who tested Pamiparib combined with proton therapy in mice with medulloblastoma [170]. Specifically, mice treated with this combination had a median survival of 96 days *versus* 85 days for those receiving only proton treatment, which aligns with the results of the previous study, although in a different tumor context. Additionally, it was noted that no significant adverse effects on normal tissue were observed, suggesting that this therapy approach may selectively sensitize tumor cells while sparing healthy tissue.

Multiple studies have directly compared the radiosensitizing effects of PARPi under X-ray and proton irradiation. In head and neck squamous cell carcinoma cell lines, L. Wang et al*.* and Zhou et al*.* reported that while PARPi in general increased sensitivity to both radiation types, with the extent of radiosensitization varying according to the experimental model and tumor site, Talazoparib exhibited superior efficacy under proton irradiation [75, 76]. Similarly, in lung and pancreatic cancer cell lines and in mice with breast cancer, Olaparib reduced cell survival more effectively after proton exposure than after X-ray exposure [46, 59, 66]. Lastly, in vivo comparison showed that pre-treatment with Olaparib did not significantly enhance radiosensitization of pancreatic cancer xenografts to X-ray irradiation, whereas a clear effect was observed after proton therapy [112]. This enhanced effect of proton therapy may be related to its higher LET, which generates more complex DNA damage primarily repaired via base excision repair, a pathway dependent on PARP activity [112]. Overall, these findings indicate that PARPi radiosensitizes tumors more effectively to proton therapy than to X-rays, although the magnitude of the effect may vary with tumor type and PARPi used.

## Carbon Ions

Carbon ions possess unique physical and biological characteristics that underpin their growing role in heavy ion therapy for cancer treatment. Their high mass and charge confer a superior mass/charge ratio, resulting in a highly localized energy deposition profile (Bragg Peak), which enables precise dose delivery to tumors while sparing surrounding healthy tissues. Compared to protons, carbon ions exhibit a sharper Bragg Peak, reduced lateral scatter, and higher ionization density, leading to improved dose conformity and minimized off-target effects [15, 16, 37, 172–176]. However, a crucial physical characteristic of carbon ions is the occurrence of nuclear interactions with the atoms of the irradiated tissue. This process, known as fragmentation, produces secondary low-energy ions. These fragments deposit their energy beyond the range of primary carbon ions, resulting in a "fragmentation tail" of dose distal to the Bragg Peak, which constitutes a physical disadvantage compared to protons [15, 16, 37]. A defining feature of carbon ions is their classification as high-LET radiation. LET values for carbon ions in the Spread-Out Bragg Peak region typically range from 10–100 keV/μm [15, 16, 37, 172, 173, 175, 177, 178]. This elevated LET is directly linked to the induction of densely clustered and complex DNA damage, which is less amenable to cellular repair mechanisms and results in enhanced tumor cell killing [15, 16, 37, 172, 175, 179, 180]. High-LET carbon ions also exhibit a reduced Oxygen Enhancement Ratio (OER), meaning their effectiveness is less dependent on tumor oxygenation status. This property is particularly advantageous for overcoming radioresistance in hypoxic tumor regions, where conventional low-LET radiation (*e.g.* photons, β-particles) is less effective [15, 16, 37, 172, 174–176]. The high LET of carbon ions translates into a high RBE, with clinical RBE values for carbon ions typically ranging from 2 to 4, compared to the fixed RBE of 1.1 for protons [15, 16, 37, 172, 174, 175, 177]. This enhanced RBE, combined with the ability to induce complex DNA lesions and reduced OER, underpins the superior efficacy of carbon ions in treating radioresistant and hypoxic tumors. These properties collectively establish carbon ions as a distinct and powerful modality within heavy ion therapy, offering both physical precision and biological potency for challenging oncological indications [15, 16, 37, 172–178, 180].

Several in vitro studies have investigated the combined effects of PARPi and carbon-ion irradiation across different tumor models, including breast [44], lung [181], pancreatic [133], glioblastoma [51], osteosarcoma [182], and chondrosarcoma [97, 183]. Among the PARPi tested, Olaparib was the most frequently used [44, 97, 133, 181–183] although other agents such as Talazoparib and Rucaparib have also been evaluated [51, 181]. Collectively, these studies consistently demonstrated that PARP inhibition enhances the cytotoxic effects of carbon-ion radiation, indicating a clear radiosensitizing effect across multiple cancer types. In particular, Hirai et al*.* proposed that this enhanced radiosensitivity arises from disruption of the DNA damage response, potentially through increased conversion of non–DSB lesions into lethal DNA damage, providing a mechanistic explanation for the observed potentiation of carbon-ion–induced cell killing [133]. Additionally, in chondrosarcoma models, Gilbert et al*.* reported no radiosensitizing effect of Olaparib in OUMS27 and JJ012 cells to carbon-ion irradiation, whereas Césaire et al*.* observed a small effect in CH2879 cells [97, 183]. This suggests that the radiosensitizing effect of PARPi may be cell-line specific, at least in certain cancer types such as chondrosarcoma.

An in vivo study in mice evaluated the effect of carbon ion radiotherapy combined with PARPi Pamiparib in medulloblastoma [170]. While carbon ions alone achieved remarkable efficacy, complete tumor regression in 79% of animals, the addition of Pamiparib did not provide a significant incremental benefit, likely due to the already potent effect of carbon ions. Neuropathological analyses confirmed the absence of detectable tumor lesions and showed that brain architecture remained indistinguishable from controls, indicating minimal adverse effects on normal tissue even after long-term follow-up. These findings suggest that carbon ion radiotherapy offers highly selective tumor targeting with a favorable safety profile, and that the role of PARPi may be more relevant for dose reduction strategies rather than enhancing efficacy in this context [170].

Additionally, several studies compared the effects of combining Talazoparib or Olaparib with distinct radiation modalities: carbon ions, X-rays and protons [44, 51, 97, 182, 183]. Overall, the combination with carbon ions generally produced greater radiosensitization than with X-rays, leading to more extensive DNA damage in glioblastoma stem cells (R633 and TG1), BRCA1-deficient breast cancer cells (HCC1937), and osteosarcoma cells (U2OS and K7M2) [44, 51, 182]. However, the results were not consistent across all tumor models. In the CH2879 chondrosarcoma cell line, the proton plus Olaparib combination produced higher cytotoxicity than carbon ion plus Olaparib, while both were more effective than X-ray plus Olaparib [97]. Conversely, in other chondrosarcoma cell lines (OUMS27 and JJ012), Olaparib treatment yielded stronger radiosensitization with X-ray than with carbon ion, the latter showing no additional effect [183]. Taken together, these findings indicate that the radiosensitizing potential of PARPi depends strongly on both the molecular characteristics of the tumor and the type of radiation used.

## Study Limitations

Importantly, this work is intended as a narrative synthesis informed by a structured literature search rather than a formal systematic review. Consequently, standardized methodological steps such as risk-of-bias assessment and meta-analysis were not performed. The evidence analyzed presents considerable heterogeneity in study design, tumor models, and treatment regimens, which limits the external validity of the findings. A major limitation concerns the variability in treatment schedules, as studies differ substantially in the timing and sequencing of PARPi administration relative to radiotherapy, including concomitant, pre-irradiation, and post-irradiation approaches. These differences may significantly influence radiosensitization outcomes, yet few studies provide direct comparisons across these regimens, making it difficult to establish the optimal schedule. Another critical issue is the absence of standardized metrics for defining or quantifying radiosensitization. The endpoints used to assess treatment efficacy vary widely, ranging from clonogenic survival and tumor growth delay to apoptosis rates and DNA damage markers, without uniform thresholds, which complicates cross-study comparisons thus hindering any quantitative integration of results through meta-analysis. Furthermore, many studies fail to clearly report radiation parameters, such as dose and fractionation schemes, or whether these align with clinical standards, hindering the extrapolation of preclinical findings to clinical practice. The diversity of tumor types and experimental models included in this review introduces additional variability in treatment response, while mechanistic insights remain inconsistent across studies. Although radiosensitization is frequently attributed to impaired DNA repair, some reports emphasize immune modulation through cGAS-STING activation, whereas others highlight replication stress or PARP trapping, and the lack of standardized mechanistic endpoints reduces clarity on the dominant pathways involved. Safety and toxicity data also remain incomplete. While early-phase clinical trials generally report manageable toxicity, preclinical studies occasionally reveal exacerbated normal tissue damage, and systematic evaluation of off-target effects and long-term toxicity is scarce, limiting risk–benefit assessment. Finally, evidence for high-LET modalities such as carbon ions and protons, as well as emerging triple-combination strategies integrating PARPi with radiotherapy and additional agents (e.g., immunotherapies or MEK inhibitors), is promising but still restricted to preclinical settings, preventing definitive conclusions on their clinical applicability. These limitations underscore the need for standardized methodologies, mechanistic validation, and rigorous clinical evaluation to optimize PARPi-based radiosensitization strategies.

## Critical View and Conclusion

**Fig. 2:**
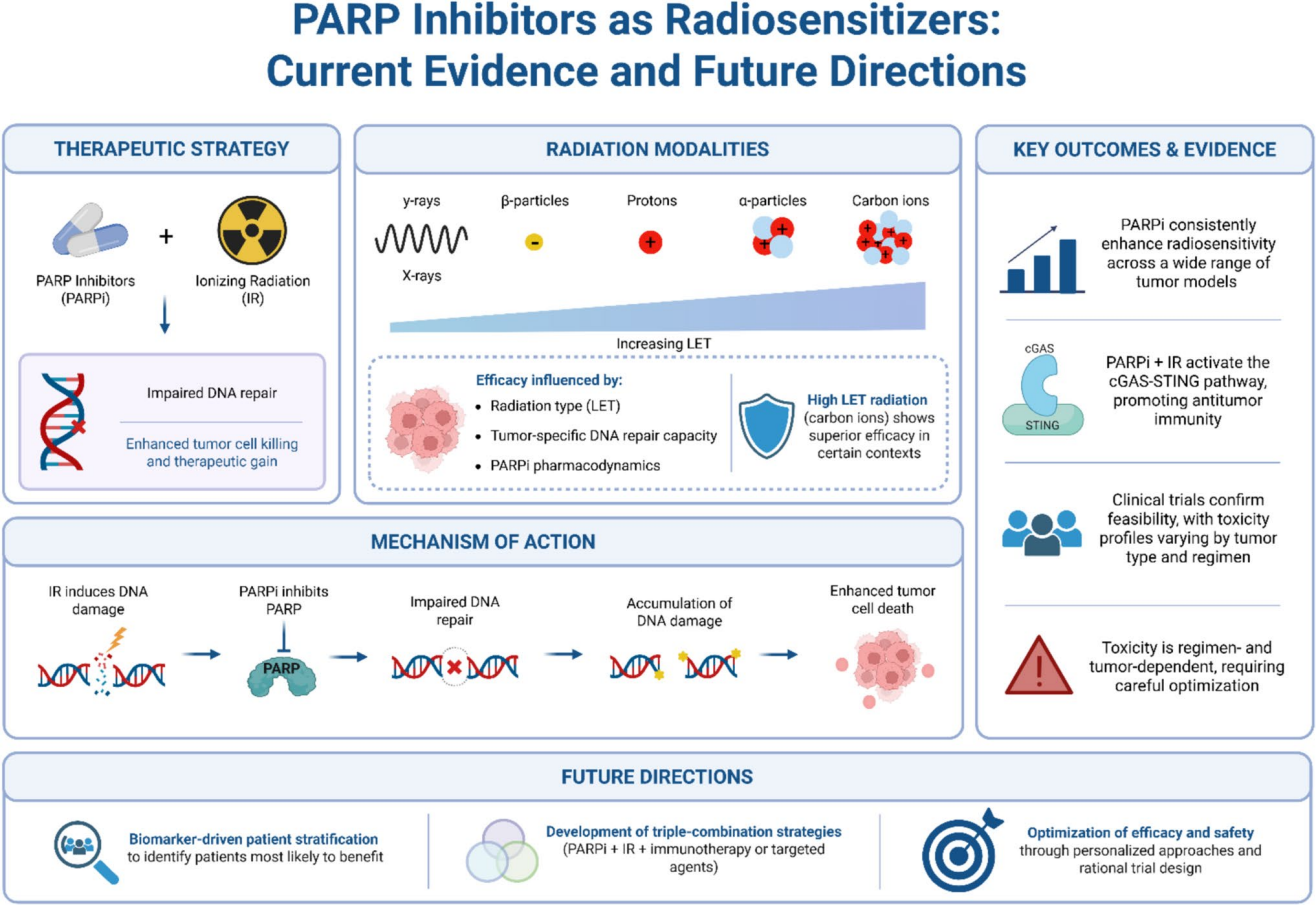
PARP Inhibitors as Radiosensitizers: Current Evidence and Future Directions. This schematic illustrates the combination of PARPi with IR as a radiosensitization strategy. PARPi impair DNA repair pathways, leading to the accumulation of DNA damage and enhanced tumor cell death following irradiation. The figure also highlights the influence of different radiation modalities, ranging from low-LET photons to high-LET particle therapy, on treatment efficacy. Key outcomes from preclinical and clinical studies are summarized, including consistent radiosensitization, activation of the cGAS–STING pathway and antitumor immunity, and variable toxicity profiles. Finally, major future directions are outlined, including biomarker-driven patient stratification, development of combination strategies, and optimization of treatment efficacy and safety. Created in BioRender. Pereira, E. (2026) https://BioRender.com/aqpdj2l

This review critically consolidates the current body of evidence supporting the use of PARPi as radiosensitizers across a broad spectrum of IR modalities and tumor types. By integrating data from 119 preclinical and clinical studies, it becomes evident that PARPi consistently enhance radiation-induced cytotoxicity, primarily through disruption of DNA damage repair pathways, while also exerting immunomodulatory effects that may amplify both local and systemic antitumor responses. Figure 2 provides an integrative overview of the therapeutic rationale, mechanisms of action, radiation modalities, key evidence, and future directions of PARPi–mediated radiosensitization. Unlike most existing reviews, which primarily emphasize photon‑based radiotherapy and address other radiation modalities only marginally, the present work deliberately adopts a radiation‑modality–oriented framework, encompassing low‑LET photons and β‑emitters as well as high‑LET modalities such as protons and carbon ions. This perspective highlights that PARPi‑mediated radiosensitization is not uniform, but instead strongly influenced by radiation quality, DNA lesion complexity, and tumor‑specific DNA repair capacity. From this synthesis, three key insights emerge: (1) PARPi consistently enhance radiosensitivity across a wide spectrum of tumor types and radiation modalities, particularly X-rays and γ-rays, by impairing DNA repair mechanisms and promoting lethal DNA damage; (2) the radiosensitizing effect of PARPi is highly dependent on tumor-specific factors, including DNA repair proficiency, mutation status, and the type of PARPi used; and (3) emerging evidence highlights the immunomodulatory potential of PARPi plus radiotherapy combinations, notably through activation of the cGAS-STING pathway and enhancement of systemic antitumor immunity.

From a critical standpoint, the accumulated evidence suggests that PARPi‑based radiosensitization has reached a level of biological maturity that justifies further clinical development, yet its translation remains uneven. While early-phase clinical trials confirm feasibility, they also expose substantial variability in toxicity profiles and therapeutic benefit, underscoring that radiosensitization cannot be dissociated from treatment context. In particular, the frequent extrapolation of PARPi doses and schedules from systemic monotherapy trials represents a conceptual limitation that may narrow the therapeutic window when combined with radiotherapy. These observations emphasize the need to move beyond empirically driven combination strategies toward biologically informed treatment design, incorporating radiation type, fractionation, spatial dose distribution, and tumor‑specific DNA repair vulnerabilities. An additional critical aspect highlighted by this review concerns safety. Accumulating evidence of enhanced gastrointestinal, skin, and hematologic toxicity demonstrates that PARP inhibition does not confer intrinsic tumor selectivity and may radiosensitize proliferative normal tissues. Consequently, the clinical utility of PARPi as radiosensitizers depends on careful therapeutic‑ratio optimization. In this regard, conformal radiotherapy and high‑LET modalities may offer opportunities to exploit PARPi‑induced radiosensitization while mitigating off‑target effects.

Looking forward, several strategic priorities emerge. These include biomarker‑driven patient stratification, systematic comparative evaluation of PARPi across radiation qualities, and the rational development of triple‑combination strategies integrating PARPi, radiotherapy, and immunomodulatory or targeted agents. Moreover, the use of PARPi as tools for radiation dose reduction, particularly in particle therapy, represents an underexplored but potentially impactful avenue to preserve tumor control while limiting toxicity.

In conclusion, PARPi constitute a biologically robust and clinically promising class of radiosensitizers. However, their successful incorporation into precision radiotherapy will require context‑aware, radiation‑specific, and biomarker‑guided implementation. By synthesizing mechanistic, preclinical, and clinical evidence within a unified radiation‑modality framework, this review not only summarizes the current state of the field but also delineates critical limitations and strategic directions necessary to advance PARPi‑based radiosensitization toward rational and individualized cancer treatment.

## Key References

- Loap P, Loirat D, Stern M-H, Pierga J-Y, Cao K, Vincent-Salomon A, et al. Safety and Potential Radiosensitizing Effect of Olaparib in Combination With Breast Radiation Therapy for Patients With Triple-Negative Breast Cancer With Residual Disease: Long-Term Results From the RADIOPARP Phase 1 Trial. Int J Radiat Oncol Biol Phys. 2025;123:726–31. 10.1016/j.ijrobp.2025.05.013.
  - ○ (Of outstanding importance) This phase I trial provides one of the strongest clinical demonstrations that olaparib can be safely combined with external beam radiotherapy in triple-negative breast cancer, with encouraging long-term outcomes. It represents a key translational step for PARPi-mediated radiosensitization in photon-based radiotherapy.
- Hu X, Zhao M, Bai M, Xue Z, Wang F, Zhu Z, et al. PARP inhibitor plus radiotherapy reshape the immune suppressive microenvironment and potentiate the efficacy of immune checkpoint inhibitors in tumors with IDH1 mutation. Cancer Lett. 2024;586:216676. 10.1016/j.canlet.2024.216676.
  - ○ (Of outstanding importance) This study supports the concept that PARPi–radiotherapy combinations extend beyond DNA repair inhibition by reshaping the tumor immune microenvironment and improving response to immune checkpoint blockade. It provides strong mechanistic and translational rationale for immunologically informed combination strategies.
- Ben Kacem M, Bright SJ, Moran E, Flint DB, Martinus DKJ, Turner BX, et al. PARP inhibition radiosensitizes BRCA1 wildtype and mutated breast cancer to proton therapy. Sci Rep. 2024;14:30897. 10.1038/s41598-024-81914-w.
  - ○ (Of importance) This preclinical work shows that PARP inhibition can enhance tumor response to proton irradiation in both BRCA-mutated and BRCA-wild-type settings, supporting the relevance of PARPi as radiosensitizers in particle therapy. It reinforces the idea that radiation quality can modulate the magnitude of PARPi-mediated radiosensitization.
- Dey P, Das R, Chatterjee S, Paul R, Ghosh U. Combined effects of carbon ion radiation and PARP inhibitor on non-small cell lung carcinoma cells: Insights into DNA repair pathways and cell death mechanisms. DNA Repair. 2024;144:103778. 10.1016/j.dnarep.2024.103778.
  - ○ (Of importance) This study provides mechanistic insight into how PARP inhibition interacts with high-LET carbon-ion irradiation, including effects on DNA repair pathway engagement and cell death programs. It supports the rationale for exploring PARPi combinations with heavy-ion therapy in selected contexts.
- Rauch H, Kitzberger C, Janghu K, Hawarihewa P, Nguyen NT, Min Y, et al. Combining [177Lu]Lu-DOTA-TOC PRRT with PARP inhibitors to enhance treatment efficacy in small cell lung cancer. Eur J Nucl Med Mol Imaging. 2024;51:4099–110. 10.1007/s00259-024-06844-1.
  - ○ (Of importance) This work demonstrates that PARP inhibition can enhance the efficacy of targeted radionuclide therapy using a β-emitter (^177^Lu), supporting the extension of PARPi radiosensitization concepts to internal radiotherapy. It highlights the therapeutic potential of PARPi in radiopharmaceutical-based strategies where DNA damage is delivered continuously at low dose rates.
- Hallqvist A, Brynjarsdóttir E, Krantz T, Sjögren M, Svensson J, Bernhardt P. 177Lu-DOTATATE in Combination with PARP Inhibitor Olaparib Is Feasible in Patients with Somatostatin-Positive Tumors: Results from the LuPARP Phase I Trial. J Nucl Med. 2025;66:707–12. 10.2967/jnumed.124.268902.
  - ○ (Of importance) This phase I study shows that combining 177Lu‑DOTATATE with olaparib is feasible, with hematologic toxicity as the principal dose‑limiting concern at higher dosing. These safety data support regimen optimization and provide a clinical foundation for further efficacy‑focused trials of PARPi plus radionuclide therapy.

## Supplementary Information

Below is the link to the electronic supplementary material.

Supplementary file1 (PDF 378 KB)

## Data Availability

No datasets were generated or analysed during the current study.

## Abbreviations

BER: Base excision repair
DSB: Double-strand break
HRR: Homologous recombination repair
IR: Ionizing radiation
LET: Linear energy transfer
NHEJ: Non-homologous end joining
OER: Oxygen enhancement ratio
PARP: Poly (ADP-ribose) polymerase
PARPi: Poly(ADP-ribose) polymerase inhibitor
PRISMA: Preferred reported items for systematic review and meta-analysis
RBE: Relative biological effectiveness
SSB: Single-strand break
