# Supplementary material for: PARP Inhibitors as Radiosensitizers: Current Evidence and Future Directions
Source: Curr Oncol Rep. 2026 Jun 1;28(1):63. doi: 10.1007/s11912-026-01800-8 (PMC13230252; doi:10.1007/s11912-026-01800-8)
Supplement: Supplementary file 1 — Supplementary file1 (PDF 378 KB) [file 11912_2026_1800_MOESM1_ESM.pdf]

Supplementary Information

PARP Inhibitors as Radiosensitizers: Current Evidence and Future Directions

Eurico Pereira[1,2,3,*], Filipa Pereira[1], Gabriela Campos[1], Ana M. Abrantes[1,2,3], Maria F. Botelho[1,2,3], Pedro Silva-Vaz[1,2,3,4], Ana S. Pires[1,2,3]

1- Univ Coimbra, Coimbra Institute for Clinical and Biomedical Research (iCBR) area of Environment Genetics and Oncobiology (CIMAGO), Institute of Biophysics, Faculty of Medicine, Azinhaga de Santa Comba, Pólo III - Pólo das Ciências da Saúde, Coimbra, 3000-548, Portugal

2- Univ Coimbra, Center for Innovative Biomedicine and Biotechnology (CIBB), Rua Larga, Coimbra, 3004-504, Portugal

3- Clinical Academic Center of Coimbra (CACC), Praceta Professor Mota Pinto, Coimbra, 3004-561, Portugal

4- Unidade Local de Saúde (ULS) de Coimbra, Centro Hospitalar e Universitário de Coimbra (CHUC), General Surgery Department, Coimbra, 3000-075, Portugal

*- Corresponding author: epereira@uc.pt

This document provides supplementary information for: PARP Inhibitors as Radiosensitizers: Current Evidence and Future Directions, Current Oncology Reports, 2026.

Methodological details

Although our review is not a systematic review, we conducted a systematic search according to guidelines proposed by the Preferred Reported Items for Systematic Review and Meta-Analysis (PRISMA) statement in order to ensure comprehensive coverage of the literature on the topic and to strengthen the robustness of our analysis [1].

On October 22, 2025, a systematic literature search was performed in Medline (via PubMed), Web of Science Core Collection, and Scopus databases, using the search queries presented in Online Resource .

Online Resource 1. Search strategy performed in Medline (via PubMed), Web of Science Core Collection, and Scopus databases.

Database	Search Query
**Medline (via PubMed)**	("Inhibitors of Poly(ADP-ribose) Polymerase*"[Title/Abstract] OR "PARP Inhibitor*"[Title/Abstract] OR "Inhibitor, PARP"[Title/Abstract] OR "Poly(ADP-ribosylation) Inhibitor*"[Title/Abstract] OR "Inhibitors, PARP"[Title/Abstract] OR "Poly(ADP-Ribose) Polymerase Inhibitor*"[Title/Abstract]) AND ("Radiotherap*"[Title/Abstract] OR "Radiation Therap*"[Title/Abstract] OR "Therapies, Radiation"[Title/Abstract] OR "Therapy, Radiation"[Title/Abstract] OR "Radiation Treatment*"[Title/Abstract] OR "Treatment, Radiation*"[Title/Abstract] OR "Treatments, Radiation*"[Title/Abstract] OR "Radiotherapy, Targeted"[Title/Abstract] OR "Radiotherapies, Targeted"[Title/Abstract] OR "Targeted Radiotherap*"[Title/Abstract] OR "Targeted Radiation Therap*"[Title/Abstract] OR "Radiation Therapies, Targeted"[Title/Abstract] OR "Therapies, Targeted Radiation*"[Title/Abstract] OR "Therapy, Targeted Radiation*"[Title/Abstract] OR "Radiation Therapy, Targeted"[Title/Abstract])

**Web of Science Core Collection**	TS=("Inhibitors of Poly(ADP-ribose) Polymerase*" OR "PARP Inhibitor*" OR "Inhibitor, PARP" OR "Poly(ADP-ribosylation) Inhibitor*" OR "Inhibitors, PARP" OR "Poly(ADP-Ribose) Polymerase Inhibitor*") AND TS=("Radiotherap*" OR "Radiation Therap*" OR "Therapies, Radiation" OR "Therapy, Radiation" OR "Radiation Treatment*" OR "Treatment, Radiation*" OR "Treatments, Radiation*" OR "Radiotherapy, Targeted" OR "Radiotherapies, Targeted" OR "Targeted Radiotherap*" OR "Targeted Radiation Therap*" OR "Radiation Therapies, Targeted" OR "Therapies, Targeted Radiation*" OR "Therapy, Targeted Radiation*" OR "Radiation Therapy, Targeted")
**Scopus**	TITLE-ABS-KEY("Inhibitors of Poly(ADP-ribose) Polymerase*" OR "PARP Inhibitor*" OR "Inhibitor, PARP" OR "Poly(ADP-ribosylation) Inhibitor*" OR "Inhibitors, PARP" OR "Poly(ADP-Ribose) Polymerase Inhibitor*") AND TITLE-ABS-KEY("Radiotherap*" OR "Radiation Therap*" OR "Therapies, Radiation" OR "Therapy, Radiation" OR "Radiation Treatment*" OR "Treatment, Radiation*" OR "Treatments, Radiation*" OR "Radiotherapy, Targeted" OR "Radiotherapies, Targeted" OR "Targeted Radiotherap*" OR "Targeted Radiation Therap*" OR "Radiation Therapies, Targeted" OR "Therapies, Targeted Radiation*" OR "Therapy, Targeted Radiation*" OR "Radiation Therapy, Targeted")

The inclusion criteria adopted were: (i) the use of at least one type of PARPi; (ii) the use of some form of radiotherapy; (iii) the use of a combination regimen of PARPi with radiotherapy; (iv) be an original article; and (v) be an article written in English, Portuguese, or Spanish. All the articles obtained from the databases were first uploaded into a reference manager software (Zotero 7.0.27) and the duplicates were removed. Then, titles and abstracts were screened based on eligibility criteria to identify potentially relevant studies. Finally, the selected articles were read in full and those that effectively met the eligibility criteria were included in this review.

Descriptive and qualitative data were extracted from each eligible reference, specifically, information pertaining to: (i) publication year; (ii) first author (iii) type of tumor; (iv) PARPi used; (v) type of radiation; (vi) type of study, *i.e. in vitro*, *in vivo* or clinical trial; (vii) and main conclusions.

The PRISMA Flow diagram presented in Online Resource 2 illustrates the selection process for the studies included in this review. The initial search resulted in 2190 articles, of which 535 were from Medline (PubMed), 691 from Web of Science Core Collection, and 964 from Scopus. After removing duplicates, 1150 papers were screened by title and abstract, resulting in 205 references. These 205 references were read in full and in the end, a total of 119 studies were included in the review. Data extracted from each selected article is gathered and summarized in Online Resource 3.

The studies were grouped and discussed according to the type of radiation, namely X-rays, γ-rays, α-particles, β-particles, protons, and carbon ions, as well as according to the type of study, *i.e. in vitro* study, *in vivo* study, or clinical trial. Concerning X-rays, 83 articles were found, of which 27 were *in vitro* studies, 6 *in vivo* studies, 41 *in vitro* and *in vivo* studies, and 9 were clinical trials. For γ-rays a total of 31 articles were found, of which 15 were *in vitro* studies, 2 *in vivo* studies, and 14 *in vitro* and *in vivo* studies. Regarding α-particles, only 1 study was found that corresponded to an *in vitro* study. Related to β-particles, 10 articles were found, of which 6 were *in vitro* studies, 1 *in vivo* study, 2 *in vitro* and *in vivo* studies, and 1 clinical trial. For protons, 11 articles were found of which 8 were *in vitro* studies, 1 *in vivo* study, and 2 *in vitro* and *in vivo* studies. Concerning carbon ions, 8 articles were found of which 7 were *in vitro* studies, and 1 was both *in vitro* and *in vivo* study.

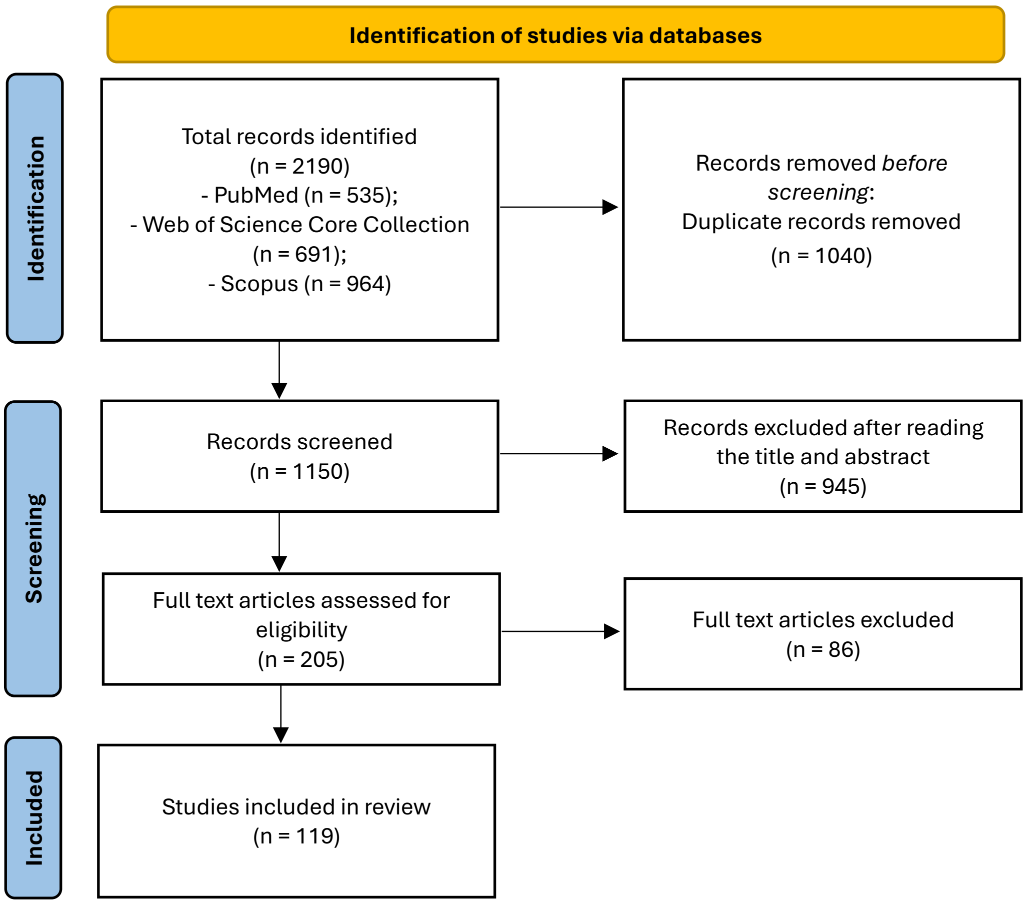

Online Resource 2. PRISMA flow diagram outlining the methodology used to conduct the systematic review, including the literature search and study selection process. Adapted from [1].

Online Resource 3. Data extracted from each selected article.

Year	First Author	Type of tumor	PARPi	Type of radiation	Type of study	Main conclusions
2025	Hallqvist *et al.* [2]	Somatostatin receptor positive tumors	Olaparib	β-particles	Clinical trial	Combining the PARPi Olaparib with ^{177}Lu-DOTATATE is feasible and generally well tolerated in patients with somatostatin receptor–positive tumors, with thrombocytopenia as the main dose-limiting toxicity.
2025	Han *et al.* [3]	Osteosarcoma, Breast cancer	Veliparib, Olaparib, Talazoparib	Protons	*In vitro*	Veliparib, Olaparib, and Talazoparib enhance radiosensitivity to high-LET proton irradiation by promoting replication stress, transcription-replication conflicts, and error-prone DNA repair.
2025	Huang *et al.* [4]	Lung cancer	Nicaraven	X-rays	*In vivo*	Nicaraven attenuated acquired radioresistance by enhancing DNA damage, apoptosis, and downregulating PARP expression, suggesting its potential as a PARPi radiosensitizer.
2025	Ijff *et al.* [5]	Cervical cancer	Olaparib	X-rays	*In vitro* and *in vivo*	Olaparib enhanced the efficacy of chemoradiotherapy and thermoradiotherapy, reducing tumor growth and increasing DNA damage and apoptosis without added toxicity.
2025	Loap *et al.* [6]	Breast cancer	Olaparib	X-rays	Clinical Trial	In patients with triple-negative breast cancer and residual disease, the combination of radiotherapy with Olaparib was safe and showed enhanced locoregional control in homologous recombination-deficient tumors.
2025	Ran *et al.* [7]	Lung cancer	Olaparib, Talazoparib, Veliparib	X-rays	*In vivo*	Olaparib, Talazoparib, and Veliparib enhanced radiosensitization and anti-PD-L1 immunotherapy efficacy by increasing chemokine expression and T cell infiltration through cGAS-STING activation and EIF4E2 downregulation.
2025	Xiao *et al.* [8]	Rectal cancer	Talazoparib	X-rays	*In vitro* and *in vivo*	In rectal cancer models, the combination of radiation with Talazoparib and the MEK inhibitor Trametinib significantly enhanced therapeutic efficacy by synergistically targeting DNA repair pathways and overcoming radiation-induced resistance.

2025	Xie *et al*. [9]	Ewing sarcoma	Talazoparib	X-rays	*In vitro* and *in vivo*	In Ewing sarcoma models, the combination of radiation therapy with Talazoparib and the topoisomerase I inhibitor Irinotecan significantly enhanced therapeutic efficacy, outperforming standard chemotherapies and demonstrating synergy driven by PARP trapping.
2025	Yang *et al*. [10]	Lung cancer	Olaparib	X-rays	*In vitro* and *in vivo*	In lung cancer models, Olaparib nanoparticles modified with Bifidobacterium-specific antibodies significantly enhanced radiosensitization by targeting hypoxic tumor regions, improving therapeutic efficacy and extending survival.
2025	Yoshino *et al*. [11]	Esophageal cancer	Nicaraven	X-rays	*In vitro*	Nicaraven exerts synthetic lethal effects and selectively enhances the cytotoxicity of X-ray irradiation in homologous recombination-deficient cancer cells, suggesting its potential as a valuable agent for combination therapy with radiotherapy.
2024	Ben Kacem *et al*. [12]	Breast cancer	Olaparib	X-rays, Protons	*In vitro* and *in vivo*	Olaparib enhances the radiosensitivity of both BRCA1-mutated and wild-type breast cancer cells to proton therapy, leading to increased DNA damage and tumor growth delay compared to photon irradiation.
2024	Brand *et al*. [13]	Esophageal cancer	AMXI-5001	X-rays	*In vitro* and *in vivo*	AMXI-5001, a dual PARP and microtubule polymerization inhibitor, potently radiosensitizes esophageal carcinoma cells and enhances tumor growth inhibition in combination with radiotherapy, outperforming clinically available PARPi.
2024	Chen *et al*. [14]	Hepatocellular cancer	Olaparib	X-rays	*In vitro* and *in vivo*	Olaparib enhances the radiosensitivity of Hepatocellular carcinoma and promotes systemic antitumor immunity via activation of the cGAS-STING pathway and chemokine signaling, leading to improved abscopal responses and efficacy of immune checkpoint blockade.
2024	Dardare *et al*. [15]	Pancreatic cancer	Olaparib	X-rays	*In vitro*	Low DDB2 expression enhances the radiosensitizing effect of Olaparib in pancreatic ductal adenocarcinoma cells by impairing DNA damage repair and promoting apoptosis, suggesting DDB2 as a predictive biomarker for radiotherapy response.

2024	Dey *et al*. [16]	Lung cancer	Olaparib, Rucaparib	Carbon ions	*In vitro*	Olaparib enhances the radiosensitivity of non-small cell lung cancer cells to carbon ion radiation by inhibiting homologous recombination and non-homologous end joining repair pathways, leading to increased apoptosis and autophagy.
2024	Dong *et al*. [17]	Osteosarcoma	Olaparib	X-rays, Carbon ions	*In vitro*	Olaparib enhances the radiosensitivity of osteosarcoma cells to X-rays and carbon ions by inducing complex DNA damage, G2/M arrest, apoptosis, and inhibiting homologous recombination repair.
2024	Durante *et al*. [18]	Ovarian cancer	Niraparib, Olaparib, Rucaparib	X-rays	Clinical trial	Stereotactic body radiotherapy combined with continued Olaparib treatment is a feasible and safe strategy that prolongs PARPi efficacy and delays systemic therapy in oligoprogressive ovarian cancer patients.
2024	Gilbert *et al*. [19]	Chondrosarcoma	Olaparib	X-rays, Carbon ions	*In vitro*	Olaparib enhances radiosensitivity of chondrosarcoma cells to X-rays but not to carbon ions, with efficacy influenced by IDH mutation status and radiation LET.
2024	Guo *et al*. [20]	Glioma	Olaparib, AZD9574	X-rays	*In vitro* and *in vivo*	Both Olaparib and AZD9574 selectively radiosensitize H3K27M-mutant diffuse midline glioma by exploiting homologous recombination deficiency and enhancing NK cell-mediated antitumor immunity, with AZD9574 showing superior therapeutic efficacy.
2024	Hu *et al*. [21]	Glioma	Veliparib	X-rays	*In vitro* and *in vivo*	In IDH1-mutant tumors, the combination of radiotherapy and the PARPi Veliparib reshapes the immunosuppressive microenvironment via cGAS-STING activation, enhancing $CD8^+$ T cell infiltration and sensitizing tumors to immune checkpoint blockade.
2024	S. Jeong *et al*. [22]	Radiation-induced intestinal damage (toxicity study on normal tissue)	Olaparib	X-rays	*In vitro* and *in vivo*	Pretreatment with Olaparib significantly exacerbated radiation-induced intestinal damage and apoptosis, indicating that its combination with radiotherapy should be used cautiously due to potential gastrointestinal toxicity.
2024	Mass *et al*. [23]	Prostate cancer	Olaparib	α-particles, β-particles	*In vitro*	Olaparib enhanced the cytotoxic effects of β-emitter ^{177}Lu, α-emitter ^{223}ra, X-rays with the strongest radiosensitizing effect observed for ^{177}Lu.

2024	Mentzel *et al.* [24]	Head and neck cancer	Talazoparib, Niraparib	X-rays	*In vitro*	Talazoparib and Niraparib could improve radiation therapy in head and neck squamous cell carcinoma. The results suggest that for PARPi, individual patient testing might be necessary due to heterogeneous responses.
2024	Navran *et al.* [25]	Head and neck cancer	Olaparib	X-rays	Clinical Trial	Olaparib at 25 mg once daily combined with conventionally fractionated radiotherapy was well tolerated and identified as the maximum tolerated dose, supporting its potential as a radiosensitizer.
2024	Rauch *et al.* [26]	Lung cancer	Olaparib, Rucaparib	β-particles	*In vitro* and *in vivo*	In small cell lung cancer models with low SSTR2 expression, the combination of ^{177}lu-DOTA-TOC with Olaparib or Rucaparib significantly enhanced therapeutic efficacy by increasing DNA damage and delaying tumor growth.
2024	Yu *et al.* [27]	Oral cancer	Olaparib	X-rays	*In vitro* and *in vivo*	Olaparib enhances radiosensitivity and inhibits metastasis by downregulating IL-17a signaling, showing synergistic effects with radiotherapy both *in vitro* and *in vivo*.
2023	Alimova *et al.* [28]	Atypical teratoid rhabdoid tumor	Rucaparib	X-rays	*In vitro* and *in vivo*	Rucaparib demonstrates significant potential as a new therapeutic strategy for atypical teratoid rhabdoid tumors by decreasing tumor growth, inducing apoptosis, and synergizing with radiation to enhance DNA damage and significantly prolong survival *in vitro* and *in vivo*.
2023	Bao *et al.* [29]	Breast cancer	Olaparib	β-particles	*In vitro* and *in vivo*	Combination of Olaparib and ^{177}Lu-DOTAGA.(SA.FAPI)$_2$ is a feasible and synergistically effective treatment strategy resulting in enhanced DNA damage and significantly prolonged overall survival with tolerable toxicity.
2023	Feijtel *et al.* [30]	Metastasized neuroendocrine tumors	Olaparib	β-particles	*In vivo*	The combination of Olaparib with β-particles resulted in a tumor-type dependent antitumor response, demonstrating enhanced efficacy in CA20948 xenografts but not in NCI-H69 xenografts, emphasizing the need for molecular stratification.
2023	Feng *et al.* [31]	Lung cancer, Cervical cancer	Niraparib	X-rays	*In vitro*	The PARPi Niraparib increases the radiosensitivity of human lung and cervical cancer cells by promoting apoptosis and inducing cell cycle redistribution, likely through inhibiting radiation-induced DNA damage repair.

2023	Palubeckaitė *et al*. [32]	Chondrosarcomas	Talazoparib	γ-rays	*In vitro*	In a spheroid model, long-term PARP inhibition with Talazoparib proved highly synergistic with Temozolomide chemotherapy across all tested IDH-status cell lines, providing a strong rationale for further preclinical exploration of the combined chemo-PARP inhibition treatment, while synergy with radiotherapy was restricted to only one cell line.
2023	Xue *et al*. [33]	Cervical cancer	Niraparib	X-rays	*In vitro* and *in vivo*	Niraparib significantly enhanced the radiosensitivity of cervical cancer xenografts by increasing DNA double-strand breaks, downregulating RAD51, and promoting apoptotic cell death.
2023	Yakovlev *et al*. [34]	Ovarian cancer	Veliparib, Olaparib	X-rays	*In vitro*	ARID1A-mutated ovarian cancer cells are highly sensitive to PARPi such as Olaparib, especially when combined with IR, due to impaired homologous recombination repair via PI3K/AKT1 pathway activation.
2023	Sheikh *et al*. [35]	Esophageal cancer	Olaparib	X-rays	Clinical trial	Olaparib combined with radiotherapy is feasible and potentially tolerable at 50 mg twice daily in patients with esophageal cancer unsuitable for conventional chemoradiotherapy, supporting further investigation of its radiosensitizing potential.
2022	Bright *et al*. [36]	Lung cancer, Pancreatic cancer	Olaparib	X-rays, Protons	*In vitro*	Targeted inhibition of DNA damage response proteins significantly radiosensitizes cancer cells to both X-ray and proton radiation, but the inconsistent efficacy across cell lines and the potential for comparable or greater radiosensitization in normal tissues critically reinforce the need for identifying predictive genetic biomarkers to guide optimal combination treatment approaches.
2022	Cui *et al*. [37]	Esophageal cancer	Niraparib	X-rays	*In vitro* and *in vivo*	Niraparib synergistically enhances the efficacy of radiation therapy against esophageal squamous cell carcinoma in preclinical models by promoting apoptosis, inhibiting proliferation, and acting via the down-regulation of FANCG expression in the FA-BRCA pathway.

2022	Delbart *et al.* [38]	Melanoma, Multiple myeloma, Pancreatic cancer, Colon cancer	Olaparib	X-rays, β-particles	*In vitro*	While X-rays and β-particles induce common radiobiological mechanisms, they differ significantly in kinetics and extent, particularly in the DNA damage response, leading to a higher radiosensitizing potential for Olaparib when combined with X-rays.
2022	Kawanishi *et al.* [39]	Breast cancer	Olaparib	X-rays, Carbon ions	*In vitro*	Carbon-ion irradiation combined with Olaparib significantly enhances cell killing in BRCA1-mutated triple-negative breast cancer cells, suggesting Olaparib as a promising radiosensitizer for carbon-ion radiotherapy.
2022	Loap *et al.* [40]	Breast cancer	Olaparib	X-rays	Clinical trial	Olaparib, when used concurrently as a radiosensitizer with radiotherapy for early-stage, high-risk triple-negative breast cancer, was safe and well tolerated up to 200 mg twice daily, warranting evaluation in larger efficacy trials.
2022	Qin *et al.* [41]	Colorectal cancer	Olaparib	X-rays	*In vitro* and *in vivo*	Olaparib significantly enhances the antitumor efficacy of radiotherapy in XRCC2-deficient colorectal cancer models by promoting persistent DNA damage, G2/M arrest, and cellular senescence, supporting its clinical use as a radiosensitizer for this specific patient subgroup.
2022	Qiu *et al.* [42]	Nasopharyngeal cancer, Lung cancer	Olaparib	X-rays	*In vitro* and *in vivo*	Olaparib synergizes with radiation therapy to sensitize tumors characterized by low ECT2 expression by counteracting sustained PARP1 activation and promoting unrepaired DNA damage, providing a viable strategy to overcome radioresistance in these specific cancer types.
2022	J. Wang *et al.* [43]	Endometrial cancer	Veliparib	X-rays	*In vitro* and *in vivo*	Veliparib significantly enhanced the radiosensitivity of endometrial carcinoma cells and xenografts by increasing DNA DSB and apoptosis.
2022	Zhou *et al.* [44]	Head and neck cancer	Olaparib, Talazoparib	X-rays, Protons	*In vitro*	PARPi Olaparib and Talazoparib enhance the radiosensitivity of 3D spheroid models of HPV-negative head and neck squamous cell carcinoma, with effectiveness correlating with homologous recombination repair deficiency and being more pronounced with Talazoparib in response to proton irradiation.

2021	Buck *et al*. [45]	Medulloblastoma	Veliparib	X-rays, γ-rays	*In vitro* and *in vivo*	Veliparib is an effective radiosensitizing agent in a preclinical model of medulloblastoma by inhibiting DNA repair and enhancing radiation-induced cytotoxicity and apoptosis, resulting in a significant increase in animal survival when combined with craniospinal irradiation.
2021	Cho *et al*. [46]	Breast cancer	Olaparib	X-rays	*In vitro* and *in vivo*	The combined treatment of Olaparib and irradiation significantly reduced tumor progression and extended survival in a preclinical mouse model.
2021	Gerossier *et al*. [47]	Hepatocellular cancer	Veliparib, Talazoparib	X-rays	*In vitro* and *in vivo*	PARPi, particularly Talazoparib, significantly potentiate radiation-induced cytotoxicity in hepatocellular carcinoma cell lines, supporting PARPi as a promising treatment strategy for hepatocellular carcinoma. This vulnerability is exacerbated in HBV-associated tumors due to HBx-mediated SMC5/6 loss, resulting in increased DNA damage levels.
2021	Jiang *et al*. [48]	Lung cancer	Olaparib	X-rays	*In vivo*	The addition of Olaparib increased the therapeutic index of hemithoracic radiation in a urethane-induced mouse lung cancer model by enhancing antitumor effects without increasing normal lung tissue toxicity.
2021	Jonuscheit *et al*. [49]	Melanoma	Talazoparib, Niraparib	X-rays	*In vitro*	Both Talazoparib and Niraparib effectively radiosensitize melanoma cells to ionizing radiation by significantly decreasing clonogenic survival and increasing G2/M arrest, demonstrating a potential therapeutic benefit over less affected healthy fibroblasts, yet emphasizing the need for prior tumor testing due to heterogeneous responses.
2021	Koosha *et al*. [50]	Glioblastoma	A-966492	β-particles	*In vitro*	The combination of β-particles with the Topoisomerase I inhibitor and the A-966492 demonstrated marked radiosensitizing effects on glioblastoma cells, suggesting an effective targeted approach to enhance radiotherapy.
2021	Kong *et al*. [51]	Lung cancer	Olaparib	X-rays, γ-rays	*In vitro* and *in vivo*	The combination of Olaparib and the BLM helicase inhibitor ML216 synergistically enhanced the radiosensitivity of Olaparib-resistant A549 non-small cell lung cancer cells, both *in vitro* and *in vivo*, by inhibiting homologous recombination repair.

2021	Romeo *et al*. [52]	Colon cancer	AZD2461	X-rays	*In vitro*	AZD2461 successfully reduced proliferation and synergistically enhanced radiosensitivity in wild-type p53 colon cancer cells by downregulating BRCA1, but was notably ineffective against cells carrying the mutant TP53.
2021	Simovic *et al*. [53]	Medulloblastoma	Pamiparib, Talazoparib	Protons, Carbon ions	*In vitro* and *in vivo*	Pamiparib significantly enhanced the survival benefit of proton irradiation in orthotopic medulloblastoma models, confirming the synthetic lethality strategy based on high HR deficiency, but yielded no statistically significant additional survival benefit when combined with the highly efficacious carbon ion radiotherapy.
2021	Waissi, Nicol, *et al*. [54]	Pancreatic cancer	Olaparib	X-rays, Protons	*In vivo*	The combination of Olaparib, Gemcitabine, and proton therapy significantly enhanced tumor control and progression-free survival in pancreatic cancer xenografts, outperforming photon-based chemoradiotherapy.
2021	Waissi, Amé, *et al*. [55]	Pancreatic cancer	Olaparib	γ-rays	*In vitro*	Olaparib significantly enhanced Gemcitabine-based chemoradiotherapy in pancreatic cancer cell lines by increasing radiosensitization, particularly at high radiation doses.
2021	N. Zhang *et al*. [56]	Lung cancer	Niraparib	X-rays	*In vitro* and *in vivo*	Niraparib acts as a radiosensitizer in EGFR-mutated non-small cell lung cancer, enhancing radiotherapy-induced antitumor immunity via activation of the STING/TBK1/IRF3 pathway and promoting $CD8^+$ T cell infiltration.
2020	Chowdhury *et al*. [57]	Lung cancer, Cervical cancer, Osteosarcoma	Olaparib	γ-rays	*In vitro*	Olaparib pretreatment prevents gamma ray-induced cell migration and metastatic potential in highly metastatic human cancer cells by inhibiting the activation of the EGFR/ERK/AKT/p38 signaling cascade and NF-kB expression.
2020	Ghorai *et al*. [58]	Glioblastoma	Olaparib	γ-rays	*In vitro* and *in vivo*	Sustained inhibition of PARP-1 activity with Olaparib enhances radiation-induced senescence in residual glioblastoma cells, significantly delaying tumor recurrence and increasing overall survival in orthotopic mouse models.

2020	Kageyama *et al.* [59]	Esophageal cancer	Olaparib	Protons	*In vitro*	Olaparib highly radiosensitizes platinum- and radiation-resistant esophageal cancer cells to multi-fractionated proton beam therapy, correlating with an additive increase in homologous recombination-dependent DNA DSB.
2020	D. Li *et al.* [60]	Cervical cancer	Olaparib	X-rays	*In vitro* and *in vivo*	Folate-conjugated active targeting Olaparib nanoparticles combined with radiotherapy significantly inhibited cervical cancer xenograft growth and prolonged survival *in vivo*, representing a promising delivery strategy for tumors overexpressing folate receptors.
2020	C. Liu *et al.* [61]	Hypopharyngeal cancer	Olaparib	X-rays	*In vitro* and *in vivo*	Olaparib significantly sensitized radioresistant hypopharyngeal carcinoma cells to radiotherapy, both *in vitro* and *in vivo*, by inhibiting PARP1 expression, suggesting a potential strategy to improve outcomes for patients with hypopharyngeal carcinoma.
2020	Tran Chau *et al.* [62]	Lung cancer	Olaparib	X-rays	*In vitro* and *in vivo*	Olaparib radiosensitized lung cancer cells *in vitro* and significantly enhanced the efficacy of fractionated radiotherapy in subcutaneous lung tumor models. However, in orthotopic lung tumors, the combination showed limited benefit and increased toxicity, indicating a narrow therapeutic window.
2020	L. Wang *et al.* [63]	Head and neck cancer	Niraparib	X-rays, Protons	*In vitro*	Niraparib enhanced the radiosensitivity of HPV-positive and HPV-negative head and neck cancer cell lines to both photon and proton irradiation, and increased proton RBE by up to 10%.
2020	Weigert *et al.* [64]	Melanoma	Niraparib, Talazoparib	X-rays	*In vitro*	Talazoparib and Niraparib enhanced radiation-induced cell death and G2/M arrest in homologous recombination-deficient melanoma cells, with variable responses across cell lines.
2019	Césaire *et al.* [65]	Chondrosarcoma	Olaparib	X-rays, Protons, Carbon ions	*In vitro*	Olaparib successfully radiosensitizes mutated chondrosarcoma cells to conventional X-rays, proton, and carbon ion irradiation, establishing a potential therapeutic strategy for this radioresistant tumor.

2019	Dubois *et al*. [66]	Breast cancer	Olaparib	X-rays	*In vitro*	Low-dose and long-term Olaparib administration synergistically enhances the efficacy of fractionated radiotherapy in triple-negative breast cancer spheroid models, often demonstrating superior potency compared to high-dose regimens.
2019	Michmerhuizen *et al*. [67]	Breast cancer	Olaparib, Veliparib	X-rays	*In vitro* and *in vivo*	Olaparib significantly radiosensitized aggressive inflammatory breast cancer models *in vitro* and *in vivo* by delaying the resolution of DNA DSB, providing the preclinical rationale for combination therapy in breast cancer patients.
2019	Carter *et al*. [68]	Colorectal cancer	Olaparib, Rucaparib, Talazoparib	X-rays, γ-rays	*In vitro* and *in vivo*	PARPi, notably Talazoparib, are highly effective radiosensitizers across BRAF-wild-type and -mutant colorectal cancer cell lines, exhibiting potentially broader efficacy than the clinical standard 5-Fluorouracil and thus warranting clinical evaluation.
2019	Ryu *et al*. [69]	Lung cancer	KJ-28d	γ-rays	*In vitro* and *in vivo*	KJ-28d demonstrates strong potential as an adjuvant therapy for BRCA-proficient non-small cell lung cancer by synergistically enhancing sensitivity to both IR and various DNA-damaging chemotherapeutic agents via the induction of increased DNA damage and reactive oxygen species.
2019	Tang *et al*. [70]	Cervical cancer	Rucaparib	X-rays	*In vitro* and *in vivo*	Rucaparib exerts significant anti-proliferative effects and serves as an effective radiosensitizer in cervical cancer both *in vitro* and *in vivo* by inducing G2/M arrest and significantly blocking IR-induced DNA DSB repair, supporting its candidacy for further clinical investigation.
2019	Venneker *et al*. [71]	Chondrosarcoma	Talazoparib	γ-rays	*In vitro*	Talazoparib sensitizes chondrosarcoma cells to Temozolomide and radiotherapy independently of IDH mutation status, suggesting a promising therapeutic strategy for unresectable tumors.
2019	Wéra *et al*. [72]	Lung cancer, Pancreatic cancer	Olaparib	X-rays, Protons	*In vitro*	Olaparib enhanced proton and X-ray radiation-induced cell death in lung and pancreatic cancer cells, especially when combined with the RAD51 inhibitor B02, demonstrating radiation-induced synthetic lethality.

2018	Bi *et al*. [73]	Ovarian cancer	Olaparib	X-rays	*In vitro* and *in vivo*	Olaparib acts as an effective radiosensitizer in high-grade serous ovarian carcinomas, providing a preclinical rationale for improved treatment modalities. Its effects are particularly more pronounced in BRCA1-deficient tumors, yet remain significant in BRCA1-proficient tumors, leading to delayed tumor growth and prolonged survival in xenograft models.
2018	Jagsi *et al*. [74]	Breast cancer	Veliparib	X-rays	Clinical trial	Concurrent Veliparib with X-rays did not exceed severe acute toxicity thresholds even at the highest tested dose (200 mg twice daily), but the high rate of severe late toxicity (grade 3 adverse events in nearly half of surviving patients at 3 years) mandates a cautious phase ii dose recommendation of 50 mg twice daily.
2018	Laird *et al*. [75]	Lung cancer	Veliparib, Talazoparib	X-rays, γ-rays	*In vitro* and *in vivo*	The PARPi Talazoparib effectively radiosensitized small cell lung cancer cell lines and xenografts to IR, demonstrating that superior PARP trapping activity enhances this therapeutic effect when compared to Veliparib.
2018	Lesueur *et al*. [76]	Glioblastoma	Talazoparib, Olaparib, AG14361	X-rays, Carbon ions	*In vitro*	Talazoparib is a promising new radiosensitizer for radioresistant glioblastoma stem cells, particularly when combined with high-LET carbon beam irradiation, which drastically reduced the Glioblastoma stem cells frequency of glioblastoma cell lines *in vitro*.
2018	Lourenco *et al*. [77]	Lung cancer	Talazoparib, Olaparib	X-rays	*In vivo*	Although PARP inhibition enhanced the antitumor response to fractionated irradiation, it also exacerbated the irradiation response in replicating normal tissues, specifically the esophagus and skin, by compromising DNA damage repair.
2018	Mao *et al*. [78]	Cholangiocarcinoma	Olaparib	X-rays	*In vitro*	Olaparib significantly enhanced the radiosensitivity of cholangiocarcinoma cells, including those without *BRCA* mutations, by inhibiting PARP1 and increasing DNA damage and apoptosis, showing promise as a novel clinical radiosensitizer for cholangiocarcinoma.

2018	Soni *et al*. [79]	Atypical Teratoid/Rhabdoid Tumor, Ewing Sarcoma, Osteosarcoma, Colorectal cancer, Lung cancer	Talazoparib, PJ34, AG14361, Olaparib, ME0328, UPF1069	X-rays	*In vitro* and *in vivo*	Talazoparib is a uniquely potent and tumor-specific radiosensitizer that achieves maximum efficacy at clinically achievable concentrations after minimal exposure times. It enhances radiation-induced cell killing by abrogating the inherent balance of DNA DSB processing, shifting repair toward error-prone alternatives and culminating in increased chromosomal translocations.
2017	Hastak *et al*. [80]	Lung cancer, Pancreatic cancer	LT626	γ-rays	*In vitro* and *in vivo*	LT626 acts as an effective radiosensitizer in lung and pancreatic cancers, synergizing with fractionated radiation to significantly decrease tumor burden and double the median survival in lung cancer xenograft models.
2017	Jue *et al*. [81]	Glioblastoma	Veliparib	X-rays	*In vitro* and *in vivo*	Veliparib effectively radiosensitizes MGMT unmethylated glioblastoma, significantly prolonging survival and inducing high levels of apoptosis in preclinical orthotopic xenograft models.
2017	Koosha *et al*. [82]	Glioblastoma	A-966492	X-rays	*In vitro*	The triple combination of A-966492, the topoisomerase I inhibitor Topotecan, and X-ray irradiation significantly enhanced radiosensitization in glioblastoma spheroids, representing a suitable strategy for improving clinical radiotherapy for glioblastoma.
2017	Rae & Mairs [83]	Prostate cancer	Olaparib, Rucaparib	X-rays	*In vitro*	Olaparib and Rucaparib significantly enhanced radiation-induced clonogenic cell kill and spheroid growth delay in prostate cancer models, demonstrating concentration-dependent radiosensitization. Both PARPi induced G_2/M arrest alone and in combination with radiation. In contrast, Elesclomol did not potentiate radiation effects, while the Hsp90 inhibitor 17-DMAG showed strong radiosensitizing activity, suggesting that targeting DNA repair and stress response pathways can improve radiotherapy efficacy in prostate cancer.

2017	Reiss *et al*. [84]	Advanced ovarian or fallopian tube cancer with peritoneal carcinomatosis	Veliparib	X-rays	Clinical trial	The phase I combination study of Veliparib and low-dose fractionated whole abdominal radiation established the maximum tolerated dose at 250mg orally twice a day. A single partial response occurred in a platinum-sensitive patient with a germline BRCA mutation, suggesting potential benefit in BRCA-mutated and/or platinum-sensitive ovarian cancers.
2017	van de Ven *et al*. [85]	Prostate cancer	Olaparib	X-rays	*In vitro* and *in vivo*	NanoOlaparib, a lipid-based injectable nanoformulation of Olaparib, successfully radiosensitizes radiation-resistant PTEN/TP53-deficient prostate tumors by enhancing intratumoral drug accumulation and local DNA damage, leading to a tripling of median survival and complete response in 50% of treated mice.
2016	Alotaibi *et al*. [86]	Colon cancer	Olaparib, Niraparib	X-rays	*In vitro*	While Olaparib and Niraparib successfully radiosensitize tumor cells by increasing DNA damage and promoting senescence, this therapeutic strategy does not interfere with the subsequent recovery of proliferative capacity in the surviving cells, thereby potentially leading to disease recurrence.
2016	Hirai *et al*. [87]	Lung cancer, Pancreatic cancer	Olaparib	Protons	*In vitro*	Olaparib effectively radiosensitizes cancer cells to proton beam irradiation, exhibiting a significantly greater enhancement ratio at the high-LET Bragg peak than at the entrance region (low-LET).
2016	Lohse *et al*. [88]	Pancreatic cancer	Olaparib	X-rays	*In vivo*	Olaparib failed to radiosensitize both *BRCA*2-mutant and –wild-type patient-derived pancreatic cancer xenografts when combined with X-rays, suggesting this combination may not be beneficial for BRCA2-related pancreatic tumors.
2016	Nile *et al*. [89]	Neuroblastoma, Glioblastoma	Rucaparib, Olaparib	X-rays, β-particles	*In vitro*	Rucaparib and Olaparib sensitize cancer cells to X-radiation or ^{131}I-MIBG treatment, effectively reducing the required radiation dose or activity concentration needed to achieve 50% cell kill, suggesting potential benefit for high-risk neuroblastoma patients.

2016	Nonnekens *et al*. [90]	Osteosarcoma	Olaparib	γ-rays, β-particles	*In vitro*	Olaparib synergistically sensitizes SSTR2-expressing tumor cells to ^{177}Lu-DOTATATE peptide receptor radionuclide therapy, significantly increasing persistent DNA DSB and cell death *in vitro* and *ex vivo*, thereby demonstrating potential to augment current peptide receptor radionuclide therapy outcomes.
2015	Chornenkyy *et al*. [91]	Astrocytoma, Glioma	Veliparib, Olaparib, Niraparib	γ-rays	*In vitro* and *in vivo*	Niraparib, identified as the most effective PARPi among those tested, acts as an effective radiosensitizer for pediatric high-grade astrocytomas and diffuse intrinsic pontine gliomas, extending survival by 60% in an orthotopic mouse model when combined with IR.
2015	Domínguez-Gómez *et al*. [92]	Breast cancer	Nicotinamide	γ-rays	*In vitro*	Nicotinamide successfully acts as a chemo- or radiosensitizing agent for breast cancer cells, enhancing the cytotoxicity of cisplatin and radiation regardless of the cells' BRCA1 expression or function.
2015	Gani *et al*. [93]	Prostate cancer	Olaparib	X-rays, γ-rays	*In vitro* and *in vivo*	Olaparib significantly radiosensitized prostate cancer *in vivo*, achieving direct tumor kill and growth delay while maintaining the therapeutic ratio by not increasing normal gut tissue toxicity under fractionated radiotherapy.
2015	Mehta *et al*. [94]	Brain metastases from primary solid tumors	Veliparib	X-rays	Clinical trial	Veliparib combined safely and tolerably with whole brain radiation therapy up to 200 mg twice daily, showing encouraging preliminary efficacy results compared to predicted survival times, supporting ongoing randomized phase 2 evaluation.
2015	Reiss *et al*. [95]	Advanced ovarian or fallopian tube cancer with peritoneal carcinomatosis	Veliparib	X-rays	Clinical trial	The final phase I study of Veliparib combined with low-dose fractionated whole abdominal radiation established the maximum tolerated dose at 250mg orally twice a day, with the single objective response observed exclusively in a germline *BRCA*-mutated, platinum-sensitive ovarian cancer patient.
2015	Verhagen *et al*. [96]	Head and neck cancer	Olaparib	γ-rays	*In vitro*	Olaparib radiosensitizes tumor cells at doses significantly lower than those required for single-agent activity, with efficacy depending on radiation dose and homologous recombination status.

2014	Guillot *et al*. [97]	Hepatocellular cancer	Veliparib	γ-rays	*In vitro* and *in vivo*	Veliparib effectively radiosensitized hepatocellular carcinoma cell lines *in vitro*, confirming the strong potential of combining PARP inhibition with radiotherapy as a therapeutic option for hepatocellular cancer.
2014	Sabbatino *et al*. [98]	Glioblastoma	NU1025	X-rays	*In vitro*	The functional status of TP53 influences the differential sensitivity of glioblastoma cells to combined treatments, rendering cells with active TP53 more sensitive to PARPi NU1025 plus radiotherapy, and cells with inactive TP53 more sensitive to NU1025 plus Topotecan.
2014	Venere *et al*. [99]	Glioblastoma	Olaparib	γ-rays	*In vitro* and *in vivo*	Olaparib sensitizes glioblastoma-initiating cells to radiation and impairs their stem cell phenotype, suggesting a promising therapeutic strategy to overcome radioresistance in glioblastoma.
2013	Chatterjee *et al*. [100]	Prostate cancer	Rucaparib	γ-rays, β-particles	*In vitro*	Rucaparib is a potent PARPi that synergistically radiosensitizes TMPRSS2-ERG fusion gene-expressing and PTEN-deficient prostate cancer cells, achieving maximal efficacy when combined with low dose-rate radiation mimicking brachytherapy.
2013	Chow *et al*. [101]	Nasopharyngeal cancer	Olaparib	γ-rays	*In vitro* and *in vivo*	Olaparib synergistically enhances the efficacy of radiotherapy and chemotherapy in nasopharyngeal carcinoma, demonstrating the therapeutic potential of targeting overexpressed PARP1.
2013	Mueller *et al*. [102]	Metastatic neuroblastoma	MK-4827	X-rays	*In vitro* and *in vivo*	Combination of MK-4827 and radiation might provide effective therapy for children with high-risk neuroblastoma.
2013	Shelton *et al*. [103]	Colorectal cancer	Veliparib	X-rays	*In vitro* and *in vivo*	Veliparib acts as a potent radiosensitizer in colorectal cancer models by inhibiting DNA double-strand break repair, demonstrating synergistic enhancement of X-rays effects when combined with Irinotecan, Oxaliplatin, or 5-Fluorouracil, and warranting clinical integration into existing chemoradiation regimens.

2012	Hirai *et al*. [104]	Pancreatic cancer	Olaparib	γ-rays, Carbon ions	*In vitro*	Olaparib significantly enhanced radiosensitivity of pancreatic cancer cells to both γ-rays and carbon-ion irradiation. Mechanistically, PARP inhibition delayed DNA damage response and DSB repair, induced S-phase arrest, and promoted subsequent G2/M arrest, suggesting that PARPi may be effective radiosensitizers for both low- and high-LET radiation therapies.
2012	Meng *et al*. [105]	Melanoma, Pancreatic cancer	Veliparib	X-rays	*In vitro* and *in vivo*	The combination of Veliparib and X-rays synergistically promotes an antitumor response mediated by $CD8^+$ T cells, achieved through the induction of senescent tumor cells with an altered immunostimulatory secretome.
2012	L. Wang *et al*. [106]	Lung cancer, Breast cancer	MK-4827	γ-rays	*In vivo*	MK-4827 significantly enhanced the efficacy of fractionated radiotherapy across multiple human tumor xenografts, independent of p53 status.
2011	Barreto-Andrade *et al*. [107]	Prostate cancer	Veliparib	X-rays	*In vitro* and *in vivo*	The efficacy of combining Veliparib with IR in prostate cancer tumors is critically dependent on the tumor's ability to undergo a competent senescence response to persistent DNA damage, which explains the disparity between *in vitro* radiosensitization and *in vivo* tumor control.
2011	Nowsheen *et al*. [108]	Head and neck cancer	Veliparib	γ-rays	*In vitro*	Veliparib significantly augments radiotherapy efficacy in head and neck cancer cells by enhancing cytotoxicity, promoting persistent DNA damage, and mechanistically reducing radiation-induced nuclear translocation of EGFR and attenuating non-homologous end-joining.
2011	Schaefer *et al*. [109]	Lymphoma	Olaparib, Veliparib	γ-rays, β-particles	*In vitro*	The PARPi Olaparib and Veliparib are highly radiosensitizing agents in lymphoma cells when combined with both external beam radiation and ^{131}I-tositumomab radioimmunotherapy, suggesting potential for selective enhancement of therapeutic efficacy in aggressive lymphoma.
2011	van Vuurden *et al*. [110]	Medulloblastoma, Ependymoma, Glioma	Olaparib	γ-rays	*In vitro*	Olaparib enhances the radiosensitivity of high-grade glioma, medulloblastoma, and ependymoma cells *in vitro*, supporting its potential as a therapeutic adjunct to radiotherapy.

2010	Khan *et al*. [111]	Head and neck cancer	GPI-15427	X-rays	*In vitro* and *in vivo*	GPI-15427 induced significant sensitization to radiotherapy in a xenograft model of human head and neck squamous cell carcinoma, representing a promising new treatment.
2009	Clarke *et al*. [112]	Glioblastoma	Veliparib	γ-rays	*In vivo*	Veliparib combined with Temozolomide and radiotherapy significantly enhanced survival in TMZ-naïve glioblastoma xenografts, while no radiosensitizing effect was observed with radiotherapy alone. In TMZ-resistant models, the addition of Veliparib provided no benefit, indicating that PARPi efficacy depends on tumor sensitivity to Temozolomide.
2009	Russo *et al*. [113]	Glioblastoma, Pancreatic cancer, Prostate cancer	E7016	X-rays	*In vitro* and *in vivo*	E7016 enhances tumor cell radiosensitivity *in vitro* by inhibiting DNA DSB repair, increasing mitotic catastrophe, and, critically, provides an enhanced tumor growth delay *in vivo* when added to the current standard of care regimen of Temozolomide and radiotherapy for glioblastoma multiforme.
2008	Dungey *et al*. [114]	Glioblastoma	Olaparib	X-rays	*In vitro*	Olaparib increases the radiosensitivity of human glioblastoma cells in a replication-dependent manner, mediated by the generation of persistent DNA DSB at collapsed replication forks, an effect enhanced by fractionation, thus improving the therapeutic ratio for glioblastoma multiforme.
2007	Albert *et al*. [115]	Lung cancer	Veliparib	γ-rays	*In vitro* and *in vivo*	Veliparib is an effective means of enhancing tumor sensitivity to radiation in lung cancer models, working by inhibiting DNA repair, promoting apoptosis and autophagy, and targeting tumor angiogenesis, thereby significantly improving tumor growth delay and showing promise for future clinical studies as a radiation sensitizer.
2004	Brock *et al*. [116]	Sarcoma	INO-1001	γ-rays	*In vitro*	INO-1001 significantly radiosensitizes sarcoma cell lines by inhibiting DNA repair mechanisms at non-cytotoxic concentrations, demonstrating high enhancement ratios, especially when combined with fractionated radiation treatments.

2004	Calabrese *et al*. [117]	Lung cancer, colorectal cancer	AG14361	X-rays, γ-rays	*In vitro* and *in vivo*	The study suggests that PARP-1 inhibition could be a promising therapeutic treatment for cancer, targeting the DNA-damage response of cancer cells, enhancing chemotherapy and radiation therapy.
2004	Chalmers *et al*. [118]	Glioblastoma	3-AB, NU1025, 4-ANI, PJ34	X-rays	*In vitro*	Low-dose radiosensitization of actively dividing tumor cells by PARP-1 inhibitors suggests their potential role in enhancing the efficacy of ultrafractionated or low-dose-rate radiotherapy regimens.
1999	Schlicker *et al*. [119]	Prostate cancer	3-ABA, ANI	γ-rays	*In vitro*	ANI acts as an effective radiosensitizer, demonstrating 1000-fold greater inhibitory potency than 3-ABA at non-toxic concentrations and suppressing post-irradiation repair processes.
1997	Weltin *et al*. [120]	Lymphoma	6(5H)-Phenanthridinone (Phen) and 3-AB	γ-rays	*In vitro*	The PARPi 6(5H)-phenanthridinone significantly enhanced radiation-induced growth inhibition, G2/M arrest, and apoptosis in lymphoma cells, suggesting its potential as a radiosensitizer.

References

1. Page MJ, McKenzie JE, Bossuyt PM, Boutron I, Hoffmann TC, Mulrow CD, et al. The PRISMA 2020 statement: an updated guideline for reporting systematic reviews. BMJ. 2021;n71. https://doi.org/10.1136/bmj.n71

2. Hallqvist A, Brynjarsdóttir E, Krantz T, Sjögren M, Svensson J, Bernhardt P. 177Lu-DOTATATE in Combination with PARP Inhibitor Olaparib Is Feasible in Patients with Somatostatin-Positive Tumors: Results from the LuPARP Phase I Trial. J Nucl Med. 2025;66:707–12. https://doi.org/10.2967/jnumed.124.268902

3. Han Y, Zhou Q, Dibbs A, Chen B, Weiskittel TM, Remmes NB, et al. Poly ADP Ribose Polymerase Inhibitors Potentiate Proton Therapy End-of-Range Effects by Accelerating Replication Forks and Promoting Transcription Conflict. Int J Radiat Oncol Biol Phys. 2025;S0360-3016(25)06197-8. https://doi.org/10.1016/j.ijrobp.2025.08.049

4. Huang K, Yan C, Abdelghany L, Zhang X, Jingu K, Li T-S. Nicaraven attenuates the acquired radioresistance of established tumors in mouse models via PARP inhibition. Mol Cell Biochem. 2025;480:341–53. https://doi.org/10.1007/s11010-024-04958-6

5. Ijff M, Mei X, Scutigliani E, Rodermond H, van Bochove G, Krawczyk P, et al. Addition of PARP1-inhibition enhances chemoradiotherapy and thermoradiotherapy when treating cervical cancer in an in vivo mouse model. Int J Hyperthermia. 2025;42. https://doi.org/10.1080/02656736.2025.2450514

6. Loap P, Loirat D, Stern M-H, Pierga J-Y, Cao K, Vincent-Salomon A, et al. Safety and Potential Radiosensitizing Effect of Olaparib in Combination With Breast Radiation Therapy for Patients With Triple-Negative Breast Cancer With Residual Disease: Long-Term Results From the RADIOPARP Phase 1 Trial. Int J Radiat Oncol Biol Phys. 2025;123:726–31. https://doi.org/10.1016/j.ijrobp.2025.05.013

7. Ran X, Wu BX, Vidhyasagar V, Song L, Zhang X, Ladak RJ, et al. PARP inhibitor radiosensitization enhances anti-PD-L1 immunotherapy through stabilizing chemokine mRNA in small cell lung cancer. Nat Commun. 2025;16:2166. https://doi.org/10.1038/s41467-025-57257-z

8. Xiao Q, Riedesser JE, Mulholland T, Li Z, Buchloh J, Albrecht P, et al. Combined MEK and PARP inhibition enhances radiation response in rectal cancer. Cell Rep Med. 2025;6:102284. https://doi.org/10.1016/j.xcrm.2025.102284

9. Xie J, Mellado-Lagarde MM, Blankenship K, Ganguly D, Twarog NR, Bianski B, et al. The Combination of PARP and Topoisomerase 1 Inhibitors Improves Radiation Therapy for Ewing Sarcoma. Cancer Sci. 2025;116:1703–14. https://doi.org/10.1111/cas.70042

10. Yang Y, Gu Y, Wu M, Zhang X, Rong J, Wu Z, et al. Radiosensitizing effects of olaparib nanoparticles modified by Bifidobacterium antibodies on lung cancer. React Funct Polym. 2025;217. https://doi.org/10.1016/j.reactfunctpolym.2025.106464

11. Yoshino Y, Ichimiya K, Jingu K, Fujita Y, Chiba N. Nicaraven enhances the cytotoxicity of X-ray irradiation in cancer cells with homologous recombination deficiency. Biochem Biophys Res Commun. 2025;742:151153. https://doi.org/10.1016/j.bbrc.2024.151153

12. Ben Kacem M, Bright SJ, Moran E, Flint DB, Martinus DKJ, Turner BX, et al. PARP inhibition radiosensitizes BRCA1 wildtype and mutated breast cancer to proton therapy. Sci Rep. 2024;14:30897. https://doi.org/10.1038/s41598-024-81914-w

13. Brand NR, Yang Y-W, Ding V, Dutta H, Peto CJ, Lemjabbar-Alaoui H, et al. Novel dual action PARP and microtubule polymerization inhibitor AMXI-5001 powerfully inhibits growth of esophageal carcinoma both alone and in combination with radiotherapy. Am J Cancer Res. 2024;14:378–89. https://doi.org/10.62347/UHLU6598

14. Chen G, Zheng D, Zhou Y, Du S, Zeng Z. Olaparib enhances radiation-induced systemic anti-tumor effects via activating STING-chemokine signaling in hepatocellular carcinoma. Cancer Lett. 2024;582:216507. https://doi.org/10.1016/j.canlet.2023.216507

15. Dardare J, Witz A, Betz M, François A, Lamy L, Husson M, et al. DDB2 expression lights the way for precision radiotherapy response in PDAC cells, with or without olaparib. Cell Death Discov. 2024;10:411. https://doi.org/10.1038/s41420-024-02188-9

16. Dey P, Das R, Chatterjee S, Paul R, Ghosh U. Combined effects of carbon ion radiation and PARP inhibitor on non-small cell lung carcinoma cells: Insights into DNA repair pathways and cell death mechanisms. DNA Repair. 2024;144:103778. https://doi.org/10.1016/j.dnarep.2024.103778

17. Dong M, Luo H, Liu R, Zhang J, Yang Z, Wang D, et al. Radiosensitization of Osteosarcoma Cells Using the PARP Inhibitor Olaparib Combined with X-rays or Carbon Ions. J CANCER. 2024;15:699–713. https://doi.org/10.7150/jca.90371

18. Durante S, Cuccia F, Rigo M, Caminiti G, Mastroleo F, Lazzari R, et al. Stereotactic radiotherapy for managing ovarian cancer oligoprogression under poly (ADP-ribose) polymerase inhibitors (PARPi). Int J Gynecol Cancer Off J Int Gynecol Cancer Soc. 2024;34:1232–9. https://doi.org/10.1136/ijgc-2024-005361

19. Gilbert A, Tudor M, Delaunay A, Leman R, Levilly J, Atkinson A, et al. Radiosensitizing Effect of PARP Inhibition on Chondrosarcoma and Chondrocyte Cells Is Dependent on Radiation LET. Biomolecules. 2024;14. https://doi.org/10.3390/biom14091071

20. Guo Y, Li Z, Parsels LA, Wang Z, Parsels JD, Dalvi A, et al. H3K27M diffuse midline glioma is homologous recombination defective and sensitized to radiotherapy and NK cell-mediated antitumor immunity by PARP inhibition. United States; 2024; https://doi.org/10.1101/2024.08.26.609803

21. Hu X, Zhao M, Bai M, Xue Z, Wang F, Zhu Z, et al. PARP inhibitor plus radiotherapy reshape the immune suppressive microenvironment and potentiate the efficacy of immune checkpoint inhibitors in tumors with IDH1 mutation. Cancer Lett. 2024;586:216676. https://doi.org/10.1016/j.canlet.2024.216676

22. Jeong S, Lee J, Park J, Son Y, Lee H, Moon C, et al. Olaparib synergically exacerbates the radiation-induced intestinal apoptosis in mice. Mol Cell Toxicol. 2024;20:979–87. https://doi.org/10.1007/s13273-023-00421-7

23. Mass A, Runge R, Wetzig K, Huebinger L, Brogsitter C, Kotzerke J. Enhancing Effects of Olaparib by Alpha- and Beta-Emitting Radionuclides, X-Rays, and Ultraviolet A Light in Combination with Ortho-IodoHoechst in a Prostate Cancer Cell Model. PHARMACEUTICALS. 2024;17. https://doi.org/10.3390/ph17111450

24. Mentzel J, Hildebrand LS, Kuhlmann L, Fietkau R, Distel LV. Effective Radiosensitization of HNSCC Cell Lines by DNA-PKcs Inhibitor AZD7648 and PARP Inhibitors Talazoparib and Niraparib. Int J Mol Sci. 2024;25. https://doi.org/10.3390/ijms25115629

25. Navran A, Al-Mamgani A, Elzinga H, Kessels R, Vens C, Tesselaar M, et al. Phase I feasibility study of Olaparib in combination with loco-regional radiotherapy in head and neck squamous cell carcinoma. Clin Transl Radiat Oncol. 2024;44:100698. https://doi.org/10.1016/j.ctro.2023.100698

26. Rauch H, Kitzberger C, Janghu K, Hawarihewa P, Nguyen NT, Min Y, et al. Combining [177Lu]Lu-DOTA-TOC PRRT with PARP inhibitors to enhance treatment efficacy in small cell lung cancer. Eur J Nucl Med Mol Imaging. 2024;51:4099–110. https://doi.org/10.1007/s00259-024-06844-1

27. Yu C-C, Lin H-Y, Chan MWY, Wu S-F, Chiou W-Y, Lee M-S, et al. Olaparib enhancing radiosensitization and anti-metastatic effect of oral cancer by targeting IL-17A signal. Cancer Cell Int. 2024;24:373. https://doi.org/10.1186/s12935-024-03547-3

28. Alimova I, Murdock G, Pierce A, Wang D, Madhavan K, Brunt B, et al. The PARP inhibitor Rucaparib synergizes with radiation to attenuate atypical teratoid rhabdoid tumor growth. Neuro-Oncol Adv. 2023;5:vdad010. https://doi.org/10.1093/noajnl/vdad010

29. Bao G, Zhou H, Zou S, Chen L, Zhang B, Wang Z, et al. Inhibition of Poly(ADP-ribose) Polymerase Sensitizes [(177)Lu]Lu-DOTAGA.(SA.FAPi)(2)-Mediated Radiotherapy in Triple-Negative Breast Cancer. Mol Pharm. 2023;20:2443–51. https://doi.org/10.1021/acs.molpharmaceut.2c01051

30. Feijtel D, Reuvers TGA, van Tuyll-van Serooskerken C, de Ridder CMA, Stuurman DC, de Blois E, et al. In Vivo Efficacy Testing of Peptide Receptor Radionuclide Therapy Radiosensitization Using Olaparib. Cancers. 2023;15. https://doi.org/10.3390/cancers15030915

31. Feng Y, Pi S, Li Y, Zhang Y, Zeng H, Xiangyu E, et al. Effects of the PARP inhibitor Niraparib on the radiosensitivity of human lung and cervical cancer cells. Radiat Med Prot. 2023;4:93–7. https://doi.org/10.1016/j.radmp.2023.05.002

32. Palubeckaitė I, Venneker S, van den Akker BEWM, Briaire-de Bruijn IH, Boveé JVMG. Does PARP Inhibition Sensitize Chondrosarcoma Cell Lines to Chemotherapy or Radiotherapy? Results From a Three-dimensional Spheroid Cell Model. Clin Orthop. 2023;481:608–19. https://doi.org/10.1097/CORR.0000000000002483

33. Xue Q, Enyang W, Tingting G, Xiaolin M, Qipeng M, Song G. Anti-tumour and radiosensitising effects of PARP inhibitor on cervical cancer xenografts. J Obstet Gynaecol J Inst Obstet Gynaecol. 2023;43:2171783. https://doi.org/10.1080/01443615.2023.2171783

34. Yakovlev VA, Sullivan SA, Fields EC, Temkin SM. PARP inhibitors in the treatment of ARID1A mutant ovarian clear cell cancer: PI3K/Akt1-dependent mechanism of synthetic lethality. Front Oncol. 2023;13:1124147. https://doi.org/10.3389/fonc.2023.1124147

35. Sheikh H, Ryder D, Bateman A, Chalmers A, Jackson A. Radiotherapy and olaparib in combination for carcinoma of the oesophagus: A phase I study. Clin Transl Radiat Oncol. 2023;40:100614. https://doi.org/10.1016/j.ctro.2023.100614

36. Bright SJ, Flint DB, Martinus DKJ, Turner BX, Manandhar M, Kacem MB, et al. Targeted Inhibition of DNA-PKcs, ATM, ATR, PARP, and Rad51 Modulate Response to X Rays and Protons. Radiat Res. 2022;198:336–46. https://doi.org/10.1667/RADE-22-00040.1

37. Cui Y, Huang W, Du F, Yin X, Feng L, Li B. Therapeutic benefits of niraparib tosylate as radio sensitizer in esophageal squamous cell carcinoma: an in vivo and in vitro preclinical study. Clin Transl Oncol. 2022;24:1643–56. https://doi.org/10.1007/s12094-022-02818-7

38. Delbart W, Karabet J, Marin G, Penninckx S, Derrien J, Ghanem G, et al. Understanding the Radiobiological Mechanisms Induced by 177Lu-DOTATATE in Comparison to External Beam Radiation Therapy. Int J Mol Sci. 2022;23. https://doi.org/10.3390/ijms232012369

39. Kawanishi M, Fujita M, Karasawa K. Combining Carbon-Ion Irradiation and PARP Inhibitor, Olaparib Efficiently Kills BRCA1-Mutated Triple-Negative Breast Cancer Cells. Breast Cancer Basic Clin Res. 2022;16:11782234221080553. https://doi.org/10.1177/11782234221080553

40. Loap P, Loirat D, Berger F, Rodrigues M, Bazire L, Pierga J-Y, et al. Concurrent Olaparib and Radiotherapy in Patients With Triple-Negative Breast Cancer: The Phase 1 Olaparib and Radiation Therapy for Triple-Negative Breast Cancer Trial. JAMA Oncol. 2022;8:1802–8. https://doi.org/10.1001/jamaoncol.2022.5074

41. Qin C, Ji Z, Zhai E, Xu K, Zhang Y, Li Q, et al. PARP inhibitor olaparib enhances the efficacy of radiotherapy on XRCC2-deficient colorectal cancer cells. Cell Death Dis. 2022;13:505. https://doi.org/10.1038/s41419-022-04967-7

42. Qiu Y, Hu W, Wen M, Zhao W, Xie J, Zhang J, et al. Low Expression of ECT2 Confers Radiation Therapy Resistance Through Transcription Coupled Nucleolar DNA Damage Repair. Int J Radiat Oncol Biol Phys. 2022;112:1229–42. https://doi.org/10.1016/j.ijrobp.2021.12.010

43. Wang J, Xing W, Lin Y, Uskenbayeva N, Yan H, Xu Y, et al. Blocking PARP activity with the inhibitor veliparib enhances radiotherapy sensitivity in endometrial carcinoma. J Clin Lab Anal. 2022;36:e24435. https://doi.org/10.1002/jcla.24435

44. Zhou C, Fabbrizi MR, Hughes JR, Grundy GJ, Parsons JL. Effectiveness of PARP inhibition in enhancing the radiosensitivity of 3D spheroids of head and neck squamous cell carcinoma. Front Oncol. 2022;12:940377. https://doi.org/10.3389/fonc.2022.940377

45. Buck J, Dyer PJC, Hii H, Carline B, Kuchibhotla M, Byrne J, et al. Veliparib Is an Effective Radiosensitizing Agent in a Preclinical Model of Medulloblastoma. Front Mol Biosci. 2021;8:633344. https://doi.org/10.3389/fmolb.2021.633344

46. Cho EJ, Kim JK, Baek HJ, Kim SE, Park EJ, Choi BK, et al. Preclinical evaluation of radiation therapy of BRCA1-associated mammary tumors using a mouse model. Int J Biol Sci. 2021;17:689–701. https://doi.org/10.7150/ijbs.53667

47. Gerossier L, Dubois A, Paturel A, Fares N, Cohen D, Merle P, et al. PARP inhibitors and radiation potentiate liver cell death in vitro. Do hepatocellular carcinomas have an achilles' heel? Clin Res Hepatol Gastroenterol. 2021;45:101553. https://doi.org/10.1016/j.clinre.2020.09.014

48. Jiang Y, Martin J, Alkadhimi M, Shigemori K, Kinchesh P, Gilchrist S, et al. Olaparib increases the therapeutic index of hemithoracic irradiation compared with hemithoracic irradiation alone in a mouse lung cancer model. Br J Cancer. 2021;124:1809–19. https://doi.org/10.1038/s41416-021-01296-y

49. Jonuscheit S, Jost T, Gajdošová F, Wrobel M, Hecht M, Fietkau R, et al. PARP Inhibitors Talazoparib and Niraparib Sensitize Melanoma Cells to Ionizing Radiation. Genes. 2021;12. https://doi.org/10.3390/genes12060849

50. Koosha F, Eynali S, Eyvazzadeh N, Kamalabadi MA. The effect of iodine-131 beta-particles in combination with A-966492 and Topotecan on radio-sensitization of glioblastoma: An in-vitro study. Appl Radiat Isot Data Instrum Methods Use Agric Ind Med. 2021;177:109904. https://doi.org/10.1016/j.apradiso.2021.109904

51. Kong Y, Xu C, Sun X, Sun H, Zhao X, He N, et al. BLM helicase inhibition synergizes with PARP inhibition to improve the radiosensitivity of olaparib resistant non-small cell lung cancer cells by inhibiting homologous recombination repair. Cancer Biol Med. 2021;19:1150–71. https://doi.org/10.20892/j.issn.2095-3941.2021.0178

52. Romeo MA, Gilardini Montani MS, Benedetti R, Arena A, Maretto M, Bassetti E, et al. Anticancer effect of AZD2461 PARP inhibitor against colon cancer cells carrying wt or dysfunctional p53. Exp Cell Res [Internet]. 2021;408. https://doi.org/10.1016/j.yexcr.2021.112879

53. Simovic M, Bolkestein M, Moustafa M, Wong JKL, Körber V, Benedetto S, et al. Carbon ion radiotherapy eradicates medulloblastomas with chromothripsis in an orthotopic Li-Fraumeni patient-derived mouse model. Neuro-Oncol. 2021;23:2028–41. https://doi.org/10.1093/neuonc/noab127

54. Waissi W, Nicol A, Jung M, Rousseau M, Jarnet D, Noel G, et al. Radiosensitizing Pancreatic Cancer with PARP Inhibitor and Gemcitabine: An In Vivo and a Whole-Transcriptome Analysis after Proton or Photon Irradiation. Cancers. 2021;13. https://doi.org/10.3390/cancers13030527

55. Waissi W, Amé J-C, Mura C, Noël G, Burckel H. Gemcitabine-Based Chemoradiotherapy Enhanced by a PARP Inhibitor in Pancreatic Cancer Cell Lines. Int J Mol Sci. Multidisciplinary Digital Publishing Institute; 2021;22:6825. https://doi.org/10.3390/ijms22136825

56. Zhang N, Gao Y, Zeng Z, Luo Y, Jiang X, Zhang J, et al. PARP inhibitor niraparib as a radiosensitizer promotes antitumor immunity of radiotherapy in EGFR-mutated non-small cell lung cancer. Clin Transl Oncol Off Publ Fed Span Oncol Soc Natl Cancer Inst Mex. 2021;23:1827–37. https://doi.org/10.1007/s12094-021-02591-z

57. Chowdhury P, Dey P, De D, Ghosh U. Gamma ray-induced in vitro cell migration via EGFR/ERK/Akt/p38 activation is prevented by olaparib pretreatment. Int J Radiat Biol. 2020;96:651–60. https://doi.org/10.1080/09553002.2020.1711461

58. Ghorai A, Mahaddalkar T, Thorat R, Dutt S. Sustained inhibition of PARP-1 activity delays glioblastoma recurrence by enhancing radiation-induced senescence. Cancer Lett. 2020;490:44–53. https://doi.org/10.1016/j.canlet.2020.06.023

59. Kageyama S, Junyan D, Hojo H, Motegi A, Nakamura M, Tsuchihara K, et al. PARP inhibitor olaparib sensitizes esophageal carcinoma cells to fractionated proton irradiation. J Radiat Res (Tokyo). 2020;61:177–86. https://doi.org/10.1093/jrr/rrz088

60. Li D, Hu C, Yang J, Liao Y, Chen Y, Fu SZ, et al. Enhanced Anti-Cancer Effect of Folate-Conjugated Olaparib Nanoparticles Combined with Radiotherapy in Cervical Carcinoma. Int J Nanomedicine. 2020;15:10045–58. https://doi.org/10.2147/IJN.S272730

61. Liu C, Gross N, Li Y, Li G, Wang Z, Zhong S, et al. PARP inhibitor Olaparib increases the sensitization to radiotherapy in FaDu cells. J Cell Mol Med. 2020;24:2444–50. https://doi.org/10.1111/jcmm.14929

62. Tran Chau V, Liu W, Gerbé de Thoré M, Meziani L, Mondini M, O'Connor MJ, et al. Differential therapeutic effects of PARP and ATR inhibition combined with radiotherapy in the treatment of subcutaneous versus orthotopic lung tumour models. Br J Cancer. 2020;123:762–71. https://doi.org/10.1038/s41416-020-0931-6

63. Wang L, Cao J, Wang X, Lin E, Wang Z, Li Y, et al. Proton and photon radiosensitization effects of niraparib, a PARP-1/-2 inhibitor, on human head and neck cancer cells. Head Neck. 2020;42:2244–56. https://doi.org/10.1002/hed.26155

64. Weigert V, Jost T, Hecht M, Knippertz I, Heinzerling L, Fietkau R, et al. PARP inhibitors combined with ionizing radiation induce different effects in melanoma cells and healthy fibroblasts. BMC CANCER. 2020;20. https://doi.org/10.1186/s12885-020-07190-9

65. Césaire M, Ghosh U, Austry J-B, Muller E, Cammarata FP, Guillamin M, et al. Sensitization of chondrosarcoma cells with PARP inhibitor and high-LET radiation. J Bone Oncol. 2019;17:100246. https://doi.org/10.1016/j.jbo.2019.100246

66. Dubois C, Martin F, Hassel C, Magnier F, Daumar P, Aubel C, et al. Low-Dose and Long-Term Olaparib Treatment Sensitizes MDA-MB-231 and SUM1315 Triple-Negative Breast Cancers Spheroids to Fractioned Radiotherapy. J Clin Med. 2019;9. https://doi.org/10.3390/jcm9010064

67. Michmerhuizen A, Pesch A, Moubadder L, Chandler B, Wilder-Romans K, Cameron M, et al. PARP1 Inhibition Radiosensitizes Models of Inflammatory Breast Cancer to Ionizing Radiation. Mol CANCER Ther. 2019;18:2063–73. https://doi.org/10.1158/1535-7163.MCT-19-0520

68. Carter R, Cheraghchi-Bashi A, Westhorpe A, Yu S, Shanneik Y, Seraia E, et al. Identification of anticancer drugs to radiosensitise BRAF-wild-type and mutant colorectal cancer. Cancer Biol Med. 2019;16:234–46. https://doi.org/10.20892/j.issn.2095-3941.2018.0284

69. Ryu H, Kim HJ, Song J-Y, Hwang S-G, Kim J-S, Kim J, et al. A Small Compound KJ-28d Enhances the Sensitivity of Non-Small Cell Lung Cancer to Radio- and Chemotherapy. Int J Mol Sci. 2019;20. https://doi.org/10.3390/ijms20236026

70. Tang M, Liu Q, Zhou L, Chen L, Yang X, Yu J, et al. The poly (ADP-ribose) polymerase inhibitor rucaparib suppresses proliferation and serves as an effective radiosensitizer in cervical cancer. Invest New Drugs. 2019;37:65–75. https://doi.org/10.1007/s10637-018-0616-7

71. Venneker S, Kruisselbrink AB, Briaire-de Bruijn IH, de Jong Y, van Wijnen AJ, Danen EHJ, et al. Inhibition of PARP Sensitizes Chondrosarcoma Cell Lines to Chemo- and Radiotherapy Irrespective of the IDH1 or IDH2 Mutation Status. Cancers. 2019;11. https://doi.org/10.3390/cancers11121918

72. Wéra A-C, Lobbens A, Stoyanov M, Lucas S, Michiels C. Radiation-induced synthetic lethality: combination of poly(ADP-ribose) polymerase and RAD51 inhibitors to sensitize cells to proton irradiation. Cell Cycle Georget Tex. 2019;18:1770–83. https://doi.org/10.1080/15384101.2019.1632640

73. Bi Y, Verginadis II, Dey S, Lin L, Guo L, Zheng Y, et al. Radiosensitization by the PARP inhibitor olaparib in BRCA1-proficient and deficient high-grade serous ovarian carcinomas. Gynecol Oncol. 2018;150:534–44. https://doi.org/10.1016/j.ygyno.2018.07.002

74. Jagsi R, Griffith KA, Bellon JR, Woodward WA, Horton JK, Ho A, et al. Concurrent Veliparib With Chest Wall and Nodal Radiotherapy in Patients With Inflammatory or Locoregionally Recurrent Breast Cancer: The TBCRC 024 Phase I Multicenter Study. J Clin Oncol Off J Am Soc Clin Oncol. 2018;36:1317–22. https://doi.org/10.1200/JCO.2017.77.2665

75. Laird JH, Lok BH, Ma J, Bell A, de Stanchina E, Poirier JT, et al. Talazoparib Is a Potent Radiosensitizer in Small Cell Lung Cancer Cell Lines and Xenografts. Clin Cancer Res Off J Am Assoc Cancer Res. 2018;24:5143–52. https://doi.org/10.1158/1078-0432.CCR-18-0401

76. Lesueur P, Chevalier F, El-Habr E, Junier M, Chneiweiss H, Castera L, et al. Radiosensitization Effect of Talazoparib, a Parp Inhibitor, on Glioblastoma Stem Cells Exposed to Low and High Linear Energy Transfer Radiation. Sci Rep. 2018;8. https://doi.org/10.1038/s41598-018-22022-4

77. Lourenco LM, Jiang Y, Drobnitzky N, Green M, Cahill F, Patel A, et al. PARP Inhibition Combined With Thoracic Irradiation Exacerbates Esophageal and Skin Toxicity in C57BL6 Mice. Int J Radiat Oncol Biol Phys. 2018;100:767–75. https://doi.org/10.1016/j.ijrobp.2017.10.051

78. Mao Y, Huang X, Shuang Z, Lin G, Wang J, Duan F, et al. PARP inhibitor olaparib sensitizes cholangiocarcinoma cells to radiation. Cancer Med. 2018;7:1285–96. https://doi.org/10.1002/cam4.1318

79. Soni A, Li F, Wang Y, Grabos M, Krieger LM, Chaudhary S, et al. Inhibition of Parp1 by BMN673 Effectively Sensitizes Cells to Radiotherapy by Upsetting the Balance of Repair Pathways Processing DNA Double-Strand Breaks. Mol Cancer Ther. 2018;17:2206–16. https://doi.org/10.1158/1535-7163.MCT-17-0836

80. Hastak K, Bhutra S, Parry R, Ford JM. Poly (ADP-ribose) polymerase inhibitor, an effective radiosensitizer in lung and pancreatic cancers. Oncotarget. 2017;8:26344–55. https://doi.org/10.18632/oncotarget.15464

81. Jue TR, Nozue K, Lester AJ, Joshi S, Schroder LBW, Whittaker SP, et al. Veliparib in combination with radiotherapy for the treatment of MGMT unmethylated glioblastoma. J Transl Med. 2017;15:61. https://doi.org/10.1186/s12967-017-1164-1

82. Koosha F, Neshasteh-Riz A, Takavar A, Eyvazzadeh N, Mazaheri Z, Eynali S, et al. The combination of A-966492 and Topotecan for effective radiosensitization on glioblastoma spheroids. Biochem Biophys Res Commun. 2017;491:1092–7. https://doi.org/10.1016/j.bbrc.2017.08.018

83. Rae C, Mairs RJ. Evaluation of the radiosensitizing potency of chemotherapeutic agents in prostate cancer cells. Int J Radiat Biol. 2017;93:194–203. https://doi.org/10.1080/09553002.2017.1231946

84. Reiss KA, Herman JM, Armstrong D, Zahurak M, Fyles A, Brade A, et al. A final report of a phase I study of veliparib (ABT-888) in combination with low-dose fractionated whole abdominal radiation therapy (LDFWAR) in patients with advanced solid malignancies and peritoneal carcinomatosis with a dose escalation in ovarian and fallopian tube cancers. Gynecol Oncol. 2017;144:486–90. https://doi.org/10.1016/j.ygyno.2017.01.016

85. van de Ven AL, Tangutoori S, Baldwin P, Qiao J, Gharagouzloo C, Seitzer N, et al. Nanoformulation of Olaparib Amplifies PARP Inhibition and Sensitizes PTEN/TP53-Deficient Prostate Cancer to Radiation. Mol Cancer Ther. 2017;16:1279–89. https://doi.org/10.1158/1535-7163.MCT-16-0740

86. Alotaibi M, Sharma K, Saleh T, Povirk LF, Hendrickson EA, Gewirtz DA. Radiosensitization by PARP Inhibition in DNA Repair Proficient and Deficient Tumor Cells: Proliferative Recovery in Senescent Cells. Radiat Res. 2016;185:229–45. https://doi.org/10.1667/RR14202.1

87. Hirai T, Saito S, Fujimori H, Matsushita K, Nishio T, Okayasu R, et al. Radiosensitization by PARP inhibition to proton beam irradiation in cancer cells. Biochem Biophys Res Commun. 2016;478:234–40. https://doi.org/10.1016/j.bbrc.2016.07.062

88. Lohse I, Kumareswaran R, Cao P, Pitcher B, Gallinger S, Bristow RG, et al. Effects of combined treatment with ionizing radiation and the PARP inhibitor olaparib in BRCA mutant and wild type patient-derived pancreatic cancer xenografts. PLOS ONE [Internet]. 2016;11. https://doi.org/10.1371/journal.pone.0167272

89. Nile DL, Rae C, Hyndman IJ, Gaze MN, Mairs RJ. An evaluation in vitro of PARP-1 inhibitors, rucaparib and olaparib, as radiosensitisers for the treatment of neuroblastoma. BMC Cancer [Internet]. 2016;16. https://doi.org/10.1186/s12885-016-2656-8

90. Nonnekens J, van Kranenburg M, Beerens CEMT, Suker M, Doukas M, van Eijck CHJ, et al. Potentiation of Peptide Receptor Radionuclide Therapy by the PARP Inhibitor Olaparib. Theranostics. 2016;6:1821–32. https://doi.org/10.7150/thno.15311

91. Chornenkyy Y, Agnihotri S, Yu M, Buczkowicz P, Rakopoulos P, Golbourn B, et al. Poly-ADP-ribose polymerase as a therapeutic target in pediatric diffuse intrinsic pontine glioma and pediatric high-grade astrocytoma. Mol Cancer Ther. 2015;14:2560–8. https://doi.org/10.1158/1535-7163.MCT-15-0282

92. Domínguez-Gómez G, Díaz-Chávez J, Chávez-Blanco A, Gonzalez-Fierro A, Jiménez-Salazar J, Damián-Matsumura P, et al. Nicotinamide sensitizes human breast cancer cells to the cytotoxic effects of radiation and cisplatin. Oncol Rep. 2015;33:721–8. https://doi.org/10.3892/or.2014.3661

93. Gani C, Coackley C, Kumareswaran R, Schütze C, Krause M, Zafarana G, et al. In vivo studies of the PARP inhibitor, AZD-2281, in combination with fractionated radiotherapy: An exploration of the therapeutic ratio. Radiother Oncol J Eur Soc Ther Radiol Oncol. 2015;116:486–94. https://doi.org/10.1016/j.radonc.2015.08.003

94. Mehta MP, Wang D, Wang F, Kleinberg L, Brade A, Robins HI, et al. Veliparib in combination with whole brain radiation therapy in patients with brain metastases: results of a phase 1 study. J Neurooncol. 2015;122:409–17. https://doi.org/10.1007/s11060-015-1733-1

95. Reiss KA, Herman JM, Zahurak M, Brade A, Dawson LA, Scardina A, et al. A Phase I study of veliparib (ABT-888) in combination with low-dose fractionated whole abdominal radiation therapy in patients with advanced solid malignancies and peritoneal carcinomatosis. Clin Cancer Res Off J Am Assoc Cancer Res. 2015;21:68–76. https://doi.org/10.1158/1078-0432.CCR-14-1552

96. Verhagen CVM, de Haan R, Hageman F, Oostendorp TPD, Carli ALE, O'Connor MJ, et al. Extent of radiosensitization by the PARP inhibitor olaparib depends on its dose, the radiation dose and the integrity of the homologous recombination pathway of tumor cells. Radiother Oncol J Eur Soc Ther Radiol Oncol. 2015;116:358–65. https://doi.org/10.1016/j.radonc.2015.03.028

97. Guillot C, Favaudon V, Herceg Z, Sagne C, Sauvaigo S, Merle P, et al. PARP inhibition and the radiosensitizing effects of the PARP inhibitor ABT-888 in in vitro hepatocellular carcinoma models. BMC Cancer. 2014;14:603. https://doi.org/10.1186/1471-2407-14-603

98. Sabbatino F, Fusciello C, Somma D, Pacelli R, Poudel R, Pepin D, et al. Effect of p53 activity on the sensitivity of human glioblastoma cells to PARP-1 inhibitor in combination with topoisomerase I inhibitor or radiation. Cytom Part J Int Soc Anal Cytol. 2014;85:953–61. https://doi.org/10.1002/cyto.a.22563

99. Venere M, Hamerlik P, Wu Q, Rasmussen R, Song L, Vasanji A, et al. Therapeutic targeting of constitutive PARP activation compromises stem cell phenotype and survival of glioblastoma-initiating cells. CELL DEATH Differ. 2014;21:258–69. https://doi.org/10.1038/cdd.2013.136

100. Chatterjee P, Choudhary GS, Sharma A, Singh K, Heston WD, Ciezki J, et al. PARP Inhibition Sensitizes to Low Dose-Rate Radiation TMPRSS2-ERG Fusion Gene-Expressing and PTEN-Deficient Prostate Cancer Cells. PLOS ONE [Internet]. 2013;8. https://doi.org/10.1371/journal.pone.0060408

101. Chow JPH, Man WY, Mao M, Chen H, Cheung F, Nicholls J, et al. PARP1 is overexpressed in nasopharyngeal carcinoma and its inhibition enhances radiotherapy. Mol Cancer Ther. 2013;12:2517–28. https://doi.org/10.1158/1535-7163.MCT-13-0010

102. Mueller S, Bhargava S, Molinaro AM, Yang X, Kolkowitz I, Olow A, et al. Poly (ADP-Ribose) polymerase inhibitor MK-4827 together with radiation as a novel therapy for metastatic neuroblastoma. Anticancer Res. 2013;33:755–62.

103. Shelton JW, Waxweiler TV, Landry J, Gao H, Xu Y, Wang L, et al. In vitro and in vivo enhancement of chemoradiation using the oral PARP inhibitor ABT-888 in colorectal cancer cells. Int J Radiat Oncol Biol Phys. 2013;86:469–76. https://doi.org/10.1016/j.ijrobp.2013.02.015

104. Hirai T, Shirai H, Fujimori H, Okayasu R, Sasai K, Masutani M. Radiosensitization effect of poly(ADP-ribose) polymerase inhibition in cells exposed to low and high liner energy transfer radiation. CANCER Sci. 2012;103:1045–50. https://doi.org/10.1111/j.1349-7006.2012.02268.x

105. Meng Y, Efimova EV, Hamzeh KW, Darga TE, Mauceri HJ, Fu Y-X, et al. Radiation-inducible immunotherapy for cancer: senescent tumor cells as a cancer vaccine. Mol Ther J Am Soc Gene Ther. 2012;20:1046–55. https://doi.org/10.1038/mt.2012.19

106. Wang L, Mason KA, Ang KK, Buchholz T, Valdecanas D, Mathur A, et al. MK-4827, a PARP-1/-2 inhibitor, strongly enhances response of human lung and breast cancer xenografts to radiation. Invest New Drugs. 2012;30:2113–20. https://doi.org/10.1007/s10637-011-9770-x

107. Barreto-Andrade JC, Efimova EV, Mauceri HJ, Beckett MA, Sutton HG, Darga TE, et al. Response of human prostate cancer cells and tumors to combining PARP inhibition with ionizing radiation. Mol Cancer Ther. 2011;10:1185–93. https://doi.org/10.1158/1535-7163.MCT-11-0061

108. Nowsheen S, Bonner JA, Yang ES. The poly(ADP-Ribose) polymerase inhibitor ABT-888 reduces radiation-induced nuclear EGFR and augments head and neck tumor response to radiotherapy. Radiother Oncol J Eur Soc Ther Radiol Oncol. 2011;99:331–8. https://doi.org/10.1016/j.radonc.2011.05.084

109. Schaefer NG, James E, Wahl RL. Poly(ADP-ribose) polymerase inhibitors combined with external beam and radioimmunotherapy to treat aggressive lymphoma. Nucl Med Commun. 2011;32:1046–51. https://doi.org/10.1097/MNM.0b013e32834a369b

110. van Vuurden DG, Hulleman E, Meijer OLM, Wedekind LE, Kool M, Witt H, et al. PARP inhibition sensitizes childhood high grade glioma, medulloblastoma and ependymoma to radiation. Oncotarget. 2011;2:984–96. https://doi.org/10.18632/oncotarget.362

111. Khan K, Araki K, Wang D, Li G, Li X, Zhang J, et al. Head and neck cancer radiosensitization by the novel poly(ADP-ribose) polymerase inhibitor GPI-15427. Head Neck. 2010;32:381–91. https://doi.org/10.1002/hed.21195

112. Clarke MJ, Mulligan EA, Grogan PT, Mladek AC, Carlson BL, Schroeder MA, et al. Effective sensitization of temozolomide by ABT-888 is lost with development of temozolomide resistance in glioblastoma xenograft lines. Mol Cancer Ther. 2009;8:407–14. https://doi.org/10.1158/1535-7163.MCT-08-0854

113. Russo AL, Kwon H-C, Burgan WE, Carter D, Beam K, Weizheng X, et al. In vitro and in vivo radiosensitization of glioblastoma cells by the poly (ADP-ribose) polymerase inhibitor E7016. Clin Cancer Res Off J Am Assoc Cancer Res. 2009;15:607–12. https://doi.org/10.1158/1078-0432.CCR-08-2079

114. Dungey FA, Löser DA, Chalmers AJ. Replication-Dependent Radiosensitization of Human Glioma Cells by Inhibition of Poly(ADP-Ribose) Polymerase: Mechanisms and Therapeutic Potential. Int J Radiat Oncol Biol Phys. 2008;72:1188–97. https://doi.org/10.1016/j.ijrobp.2008.07.031

115. Albert J, Cao C, Kim K, Willey C, Geng L, Xiao D, et al. Inhibition of poly (ADP-ribose) polymerase enhances cell death and improves tumor growth delay in irradiated lung cancer models. Clin CANCER Res. 2007;13:3033–42. https://doi.org/10.1158/1078-0432.CCR-06-2872

116. Brock WA, Milas L, Bergh S, Lo R, Szabo C, Mason KA. Radiosensitization of human and rodent cell lines by INO-1001, a novel inhibitor of poly(ADP-ribose) polymerase. Cancer Lett. 2004;205:155–60. https://doi.org/10.1016/j.canlet.2003.10.029

117. Calabrese CR, Almassy R, Barton S, Batey MA, Calvert AH, Canan-Koch S, et al. Anticancer chemosensitization and radiosensitization by the novel poly(ADP-ribose) polymerase-1 inhibitor AG14361. J Natl Cancer Inst. 2004;96:56–67. https://doi.org/10.1093/jnci/djh005

118. Chalmers AJ, Johnston P, Woodcock M, Joiner M, Marples B. PARP-1, PARP-2, and the cellular response to low doses of ionizing radiation. 2004;58:410–9. https://doi.org/10.1016/j.ijrobp.2003.09.053

119. Schlicker A, Peschke P, Bürkle A, Hahn EW, Kim JH. 4-Amino-1,8-naphthalimide: a novel inhibitor of poly(ADP-ribose) polymerase and radiation sensitizer. Int J Radiat Biol. 1999;75:91–100. https://doi.org/10.1080/095530099140843

120. Weltin D, Holl V, Hyun JW, Dufour P, Marchal J, Bischoff P. Effect of 6(5H)-phenanthridinone, a poly (ADP-ribose)polymerase inhibitor, and ionizing radiation on the growth of cultured lymphoma cells. Int J Radiat Biol. 1997;72:685–92. https://doi.org/10.1080/095530097142843
